# Establishment of Novel DNA Methylation-Based Prostate Cancer Subtypes and a Risk-Predicting Eight-Gene Signature

**DOI:** 10.3389/fcell.2021.639615

**Published:** 2021-02-23

**Authors:** Enchong Zhang, Fujisawa Shiori, Oscar YongNan Mu, Jieqian He, Yuntian Ge, Hongliang Wu, Mo Zhang, Yongsheng Song

**Affiliations:** ^1^Department of Urology, Shengjing Hospital of China Medical University, Shenyang, China; ^2^Department of Breast and Endocrine Surgery, Tohoku University Hospital, Sendai, Japan; ^3^PANASIAUSMLE, Association of Asian Medical Graduates, Toronto, ON, Canada; ^4^Department of Spine and Joint Surgery, Shengjing Hospital of China Medical University, Shenyang, China

**Keywords:** prostate cancer, DNA methylation, predictive signature, prognosis, *RND3*, hypermethylation, systems biology

## Abstract

Prostate cancer (PCa) is the most common malignant tumor affecting males worldwide. The substantial heterogeneity in PCa presents a major challenge with respect to molecular analyses, patient stratification, and treatment. Least absolute shrinkage and selection operator was used to select eight risk-CpG sites. Using an unsupervised clustering analysis, called consensus clustering, we found that patients with PCa could be divided into two subtypes (Methylation_H and Methylation_L) based on the DNA methylation status at these CpG sites. Differences in the epigenome, genome, transcriptome, disease status, immune cell composition, and function between the identified subtypes were explored using The Cancer Genome Atlas database. This analysis clearly revealed the risk characteristics of the Methylation_H subtype. Using a weighted correlation network analysis to select risk-related genes and least absolute shrinkage and selection operator, we constructed a prediction signature for prognosis based on the subtype classification. We further validated its effectiveness using four public datasets. The two novel PCa subtypes and risk predictive signature developed in this study may be effective indicators of prognosis.

## Introduction

As the most common cancer in males, prostate cancer (PCa) is a major public health threat (Siegel et al., 2020). Based on the latest global cancer data from the World Health Organization (WHO)^1^, the age-standardized rate for PCa ranks second, 5-year prevalence ranks first, and age-standardized mortality ranks sixth. Despite the ongoing development of therapeutic strategies, the heterogeneity of PCa contributes to treatment failure (Chang et al., 2014; Peng et al., 2018). Therefore, it is necessary to identify subtypes of PCa during diagnosis and treatment.

During the process of DNA methylation, methyl groups are added to CpG islands on the DNA molecule. Hypermethylation acts on promoters and could lead to gene silencing, whereas hypomethylation is associated with chromosomal instability and a loss of imprinting (Daura-Oller et al., 2009). In many diseases, including cancer, abnormal hypermethylation on gene promoters, which could be inherited by daughter cells, has been detected (Wang and Lei, 2018). Abnormal DNA methylation status is now considered a significant determinant of cancer development. Abundant stable DNA methylation in the genome is a candidate for diagnosis and treatment (Mikeska and Craig, 2014). Accordingly, the use of DNA methylation status to divide PCa into subtypes may provide important new insights.

Novel methylation-based subtypes have been reported in PCa. For example, the Cancer Genome Atlas Research Network (Abeshouse et al., 2015) conducted a comprehensive molecular analysis of 333 primary prostate carcinomas and identified different subtypes with highly diverse genomic, epigenomic, and transcriptomic patterns. In particular, an unsupervised hierarchical cluster analysis of the 5,000 most variable hypermethylated CpG sites revealed four epigenetically distinct subtypes of PCa. In another multicenter study, a new epigenetic CpG methylator phenotype in advanced PCa was reported, and this subtype is characterized by hypermethylation both within and outside CpG sites, shores, and shelves (Zhao et al., 2020). These important studies have improved our understanding of the DNA methylation landscape in PCa, providing a reference for future research. Recent studies have focused on revealing associations of novel DNA methylation subtypes with driver events in the genome and transcriptome. Utilizing a different approach, in this study, we identified DNA methylation subtypes based on clinical outcomes, with further analyses linking these subtypes with molecular mechanisms. In particular, we screened out eight CpG sites in which the DNA methylation status was associated with prognosis in PCa and identified two subtypes based on these DNA methylation statuses. Thereafter, differences in the epigenome, genome, transcriptome, disease status, immune cell infiltration, and function between these two subtypes were explored. Lastly, genes related to the high-risk subtype were selected and screened to construct an eight-gene signature with the ability to predict prognosis. The effectiveness of the signature was validated using four public datasets.

## Materials and Methods

### Data Processing

RNA-seq data (in the form of HTSeq-Counts and HTSeq-FPKM), DNA methylation (450 K), somatic variation, copy number alterations (CNA), and clinical information for patients with PCa were downloaded from The Cancer Genome Atlas (TCGA) database^2^ (Blum et al., 2018). The gene annotation file was downloaded from the Ensembl database^3^ (Howe et al., 2020). RNA-seq data in FPKM were converted to TPM. The TPM and β-values for CpG sites were quantile-normalized. In total, 477 patients with both the data described above (RNA-seq and methylation data) and complete clinical information were included. Disease-related clinical information for these patients is provided in Table 1. To validate the effectiveness of the risk signature, gene expression profiles and clinical data from four public datasets were used, i.e., GSE70769 and GSE116918 from the Gene Expression Omnibus (GEO) database^4^ (Barrett et al., 2013; Ross-Adams et al., 2015; Jain et al., 2018) and DKF2018 and MSKCC2010 from cBioPortal for Cancer Genomics^5^ (Cerami et al., 2012; Gao et al., 2013). Information about these four datasets is provided in Table 2.

**TABLE 1:** The disease-related clinical information of patients with PCa included in the study.

Characteristics	Value
Patients (*n*)	477
Age (year), median (IQR)	62.0 (56.0–66.0)
PSA (ng/ml), median (IQR)	7.5 (5.1–11.4)
**Pathological Gleason score**, ***n* (%)**	
≤6	43 (9.0%)
7 (3+4)	143 (30.0%)
7 (4+3)	100 (21.0%)
8	56 (11.7%)
9∼10	135 (28.3%)
**Prior malignancy**, ***n* (%)**	
No	450 (94.3%)
Yes	27 (5.7%)
**Race**, ***n* (%)**	
Asian	12 (2.5%)
Whit, American Indian or Alaska native	398 (83.4%)
Black or African American	55 (11.6%)
NA	12 (2.5%)
**Residual tumor**, ***n* (%)**	
R0	301 (63.1%)
R1	15 (3.1%)
R2	142 (29.8%)
Rx	5 (1.0%)
NA	14 (3.0%)
**Clinical M**, ***n* (%)**	
M0	437 (91.6%)
M1a or M1c	2 (0.4%)
NA	38 (8.0%)
**Pathological T**, ***n* (%)**	
T1c	2 (0.4%)
T2a	13 (2.7%)
T2b	10 (2.1%)
T2c	160 (33.5%)
T3a	151 (31.7%)
T3b	129 (27.0%)
T4	9 (1.9%)
NA	3 (0.7%)
**Pathological N**, ***n* (%)**	
N0	329 (69.0%)
N1	78 (16.4%)
NA	70 (14.6%)
**Diagnostic CT or MRI**, ***n* (%)**	
No evidence of extraprostatic extension	196 (41.1%)
Equivocal	6 (1.3%)
Extraprostatic extension localized	22 (4.6%)
Extraprostatic extension	9 (1.9%)
NA	244 (51.1%)
**Outcome**, ***n* (%)**	
DFS	53 (11.1%)
Disease free	424 (88.9%)

*PCa, prostate cancer; PSA, prostate-specific antigen; DFS, disease-free survival; IQR, interquartile range; NA, not analyzed.*

**TABLE 2:** Information of the four publicly available independent validation datasets.

Dataset	Sample size	Transcriptome platform	Tissue
DKFZ2018	82	Illumina HiSeq 2000 (RNAseq)	Fresh frozen
GSE70769	90	Illumina humanHT-12 V4.0	Fresh frozen
GSE1166918	248	ADXPCv1a520642 Affymetrix human	Formalin-fixed Paraffin-embedded
MSKCC2010	140	Affymetrix human Exon 1.0 ST array	Fresh frozen

### Identification of DNA Methylation-Based Subtypes

We previously identified 120 CpG sites that were differentially methylated between PCa and normal prostate tissues and were significantly associated with disease-free survival (DFS) (Zhang et al., 2020b). Least absolute shrinkage and selection operator (LASSO) regression enables variable selection and regularization, while fitting the generalized linear model. Therefore, LASSO regression was used to reduce the number of CpG sites as the input for subtype identification using the glmnet R package (Engebretsen and Bohlin, 2019). After the CpG sites were screened, the β values for these sites in 477 patients were used as inputs for consensus clustering, an unsupervised clustering analysis. Consensus clustering was performed using the ConsensusClusterPlus R package (Monti et al., 2003) and the following operating parameters: maxK = 10, reps = 1000, pItem = 0.8, pFeature = 1, clusterAlg = hc, and distance = pearson. A heatmap was generated to visualize the methylation status of the subtypes using the pheatmap R package (Kolde and Kolde, 2015). Furthermore, a survival analysis of the subtypes was performed by the log rank test using the survival R package (Therneau and Lumley, 2014). To further demonstrate the differences between subtypes, a principal component analysis was performed using the 477 patients. Finally, correlations between disease-related clinical information and subtype status were evaluated by the Mann–Whitney *U* test, χ^2^ test, or Fisher’s exact test.

### Single Nucleotide Variation Between Subtypes

Simple nucleotide variants were compared between subtypes using the GenVisR R package (Skidmore et al., 2016). Genes with the top 10 mutation frequencies were displayed in a waterfall plot. According to the results of the waterfall plot, the difference between the mRNA levels of mutant and wild-type Speckle-type POZ Protein (*SPOP*) was evaluated. Then, the mRNA levels of *SPOP* in different subtypes were compared by Wilcoxon’s test.

### Differences in Copy Number Alterations, TMPRSS2–ERG Fusion, and Androgen Receptor Scores Between Subtypes

To explore the difference in CNAs between subtypes, genes with significant differences in copy number between subtypes were identified by chi-squared tests. Among these genes, *RND3* was differentially expressed between the subtypes, as determined by a Wilcoxon test. The type and frequency of CNAs in *RND3* were explored. Furthermore, the relationship between CNA types and mRNA expression levels of *RND3* were evaluated by the Wilcoxon test.

Data from The Tumor Fusion Gene Data Portal database (https://www.tumorfusions.org/) were used to analyze the difference in *TMPRSS2–ERG* fusion gene expression between the subtypes (Yoshihara et al., 2015). Finally, the androgen receptor (AR) score in each subtype was compared by the Wilcoxon test. AR scores were obtained from the cBioPortal database (Cancer Genome Atlas Research Network, 2015).

### Immune Cells in the Tumor Microenvironment in Each Subtype

RNA-seq data in TPM format were uploaded to CIBERSORTx^6^ (Newman et al., 2019) to evaluate the infiltration of 22 types of immune cells in the tumor microenvironment. The abundances of these immune cells were compared between subtypes using a violin plot and the Wilcoxon test. Furthermore, survival curves were generated for these cells. The p-values for the survival analysis were calculated by a Cox regression and log-rank test using the survival R package (Therneau and Lumley, 2014).

### Functional Enrichment Analysis of Subtypes

Fold change values for gene expression differences between the subtypes were used as the ranks in a gene set enrichment analysis (GSEA). To obtain fold changes, HTSeq-Counts were analyzed using the DESeq2 R package (Love et al., 2014). The hallmark gene set downloaded from the Molecular Signatures Database9 v7.1^7^ was used as the reference gene list in the GSEA (Subramanian et al., 2005; Liberzon et al., 2011). Finally, the GSEA was completed using the clusterProfiler R package (Yu et al., 2012).

Furthermore, expression levels of genes that were crucial for PCa were compared between the subtypes by Wilcoxon tests.

### Weighted Correlation Network Analysis of Subtypes

A weighted correlation network analysis (WGCNA) could be used find phenotype-associated gene modules (Langfelder and Horvath, 2008; Li et al., 2019). Therefore, TPM values from RNA-seq data were used as the input for a WGCNA. Eight was the soft power threshold to construct a network that simultaneously satisfied a scale-free topology and high connectivity. Pearson correlation coefficients for the relationships between phenotypes and gene modules were determined. The phenotypes included PSA (prostate-specific antigen), Gleason score, and the subtypes. The gene module most closely associated with the high-risk phenotypes was identified. Differentially expressed genes (DEGs) between PCa and normal prostate tissues in the selected gene module were identified. The conditions for DEGs were logarithmic fold changes (| LFCs|) > 1 and *p* < 0.05. Then, survival-associated genes were screened from the DEGs by Cox regression and log-rank tests. Finally, genes for LASSO were filtered out.

### Identification of a Risk Signature in the Training Set

Before training, 477 patients were randomly divided into a training set and internal validation set using the caret R package (Kuhn, 2008). Information for patients in the training set is provided in Supplementary Table 1 and information for the internal validation set is provided in Supplementary Table 2. LASSO regression was used to construct a single signature for predicting prognosis with high performance (Svane et al., 2018). LASSO regression was applied to the training set; during the selection of genes, the C-index after 10-fold cross-validation reflected the effect of different screening strategies. Genes with the maximal C-index values were selected for the prognostic signature using the glmnet R package (Engebretsen and Bohlin, 2019) with the following parameter settings: family = Cox, type.measure = C, parallel = TRUE, with default settings for other parameters. Furthermore, the difference in the risk score between subtypes identified and the relationship between the risk score and survival were evaluated. Comparisons were performed using the Wilcoxon test.

### Predictive Accuracy of the Signature

First, time-dependent receiver operating characteristic (tdROC) curves were used to evaluate the predictive accuracy of the signature in the training set, internal validation set, and external validation sets (DKFZ2018, GSE70769, GSE116918, and MSKCC2010) using the timeROC R package (Blanche and Blanche, 2013). Then, a survival analysis by Cox regression and the log-rank test was performed using these datasets. Survival curves were plotted using the Kaplan–Meier method and the survminer R package (Kassambara et al., 2017).

Univariate and multivariate Cox regression analyses were used to explore whether the risk score is an independent predictor of prognosis. Finally, the clinical diagnostic value of the signature was compared with that of clinical features (Gleason score and PSA) by a decision curve analysis (DCA) (Van Calster et al., 2018). DCA is used to compare prediction models that incorporate clinical outcomes; it requires only the dataset on which the models are tested and can be applied to models with either continuous or dichotomous results (Zhang et al., 2020a).

### Statistical Analysis

R 3.6.3 was used for all statistical analyses. Values of *p* < 0.05 were defined as statistically significant. In the survival analysis, the survival outcome was defined as DFS or biochemical recurrence-free survival (BCR) based on clinical records.

## Results

### Identification of Two DNA Methylation-Based Subtypes

**FIGURE 1:**
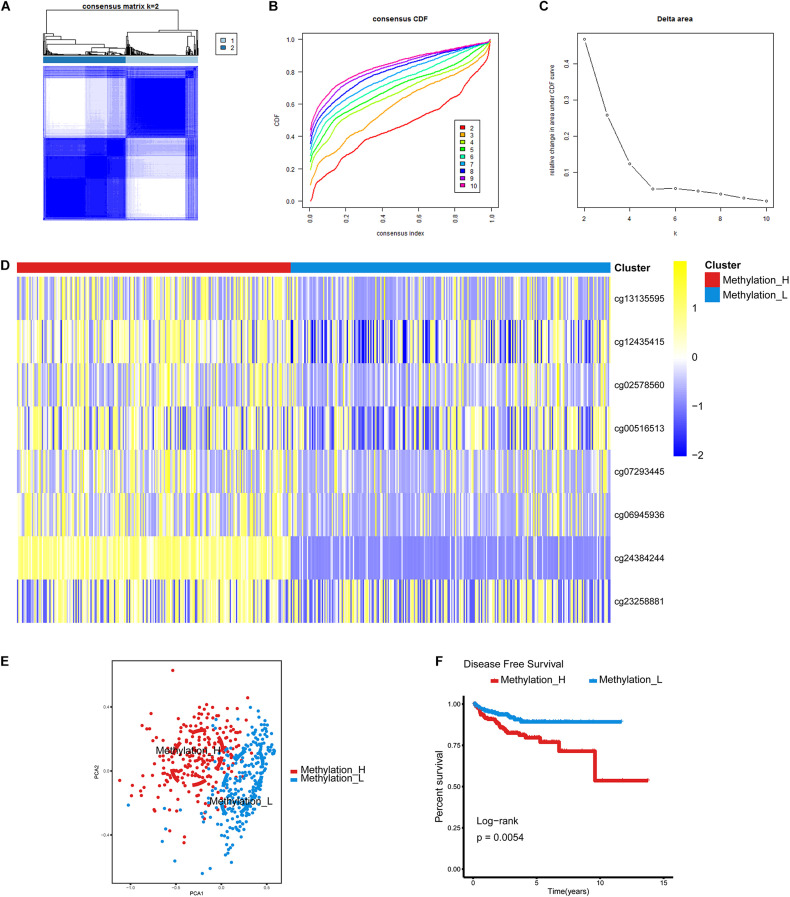
Identification of two DNA-methylation-based subtypes. **(A)** The consensus matrix obtained when *k* = 2. Consistency values range from 0 to 1, 0 means never clustering together (white), one means always clustering together (dark blue). **(B)** The CDF curve under different values of k. The value of k represents the number of clusters during the consensus cluster. When the optimal k value is reached, the area under the CDF curve will not significantly increase with the increase of k value. **(C)** Relative change in area under CDF curve under different values of *k*. **(D)** Differences between the statuses of DNA methylation of the two subtypes. **(E)** PCA showed that patients in the different subtypes were significantly different from each other. **(F)** Survival curves for patients in the different subtypes (PCa, prostate cancer; CDF, cumulative distribution function; PCA, principal components analysis. And *p* < 0.05 was defined as statistically significant).

The cumulative distribution function (CDF) and relative change in the area under the CDF curve are shown in Figures 1A,B, respectively. According to Monti et al. (2003), the optimal k value is determined by a number of factors. One of the criteria is that when the optimal k value is reached, the area under the CDF curve will not increase significantly with increases in k. Therefore we first assumed that the optimal value in this study was set to *k* = 5, indicating that the cohort could be divided into up to five subtypes. However, one cluster consisted of only a single patient when *k* = 4 or 5. Additionally, the cluster-consensus value for each cluster was not large enough under *k* = 4 or 5 (Supplementary Figure 1). Therefore, we focused on *k* = 2 or 3. For *k* = 3, patients in C1 showed a worse prognosis, and patients in C2 and C3 did not show an obvious difference in prognosis (Figure 1C). For *k* = 2, patients in Methylation_H had worse a prognosis than that of patients in Methylation_L (Figure 1D). Furthermore, the Methylation_H subtype and C1 subtype included the same patients and the Methylation_L subtype consisted of patients in the C2 and C3 subtypes. Groups for different k values are shown in Supplementary Table 3. Ultimately, we identified two subtypes with a difference in prognosis. The consensus matrix is displayed in the form of a heatmap in Figure 1E and the consensus clustering analysis is summarized in Supplementary Figure 1. A principal component analysis revealed clear separation between the Methylation_H subtype and the Methylation_L subtype (Figure 1F). As shown in Figure 1G, these two subtypes had different levels of DNA methylation at CpG sites. We defined the hypermethylated subtype as Methylation_H and the hypo methylated subtype as Methylation_L. As shown in Table 3, age, PSA, Gleason score, residual tumor status, pathological T, and clinical outcome of patients between Methylation_H and Methylation_L subtypes are different significantly.

**TABLE 3:** The association between subtypes and disease-related clinical information of PCa.

Clinicopathologic variables	Consensus clusters		*P*
	Methylation_H (*n* = 220)	Methylation_L (*n* = 257)	
Age (year), median (IQR)	63.0 (57.8–67.0)	60.0 (55.0–65.0)	0.001a
PSA (ng/ml), median (IQR)	8.0 (5.8–12.9)	6.8 (4.6–10.1)	< 0.001a
Pathological Gleason score, *n* (%)			< 0.001b
≤6	11 (5.0%)	32 (12.5%)	
7 (3+4)	53 (24.1%)	90 (35.0%)	
7 (4+3)	45 (20.5%)	55 (21.4%)	
8	26 (11.8%)	30 (11.7%)	
9∼10	85 (38.6%)	50 (19.4%)	
Prior malignancy, *n* (%)			0.828b
No	207 (94.1%)	243 (94.6%)	
Yes	13 (5.9%)	14 (5.4%)	
Race, *n* (%)			0.529b
Asian	7 (3.2%)	5 (2.0%)	
Whit, American Indian or Alaska native	181 (82.3%)	217 (84.4%)	
Black or African American	28 (12.7%)	27 (10.5%)	
NA	4 (1.8%)	8 (3.1%)	
Residual tumor, *n* (%)			
R0	126 (57.3%)	175 (68.1%)	0.005b
Rx/R1/R2	90 (40.9%)	72 (28.0%)	
NA	4 (3.0%)	10 (3.9%)	
Clinical M, *n* (%)			0.226c
M0	207 (94.1%)	230 (89.5%)	
M1a or M1c	2 (0.9%)	0 (0.0%)	
NA	11 (5.0%)	27 (10.5%)	
Pathological T, *n* (%)			0.013c
T1c	0 (0.0%)	2 (0.8%)	
T2a	3 (1.4%)	10 (3.9%)	
T2b	3 (1.4%)	7 (2.7%)	
T2c	63 (28.6%)	97 (37.7%)	
T3a	71 (32.3%)	80 (31.1%)	
T3b	73 (33.2%)	56 (21.8%)	
T4	6 (2.7%)	3 (1.2%)	
NA	1 (0.4%)	2 (0.8%)	
Pathological N, *n* (%)			0.289b
N0	151 (68.6%)	178 (69.3%)	
N1	41 (18.6%)	37 (14.4%)	
NA	28 (12.8%)	42 (16.3%)	
Diagnostic CT or MRI, *n* (%)			
No evidence of extraprostatic extension	95 (43.2%)	101 (39.3%)	0.135c
Equivocal	5 (2.3%)	1 (0.4%)	
Extraprostatic extension localized	10 (4.5%)	12 (4.7%)	
Extraprostatic extension	7 (3.2%)	2 (0.8%)	
NA	103 (46.8%)	141 (54.8%)	
Outcome, *n* (%)			
DFS	35 (8.3%)	18 (7.0%)	0.002b
Disease free	185 (91.7%)	239 (93.0%)	

*P values were calculated by the Mann–Whitney U test (a), Chi-square test (b), or Fisher’s exact test (c). PCa, prostate cancer; PSA, prostate-specific antigen; DFS, disease-free survival; IQR, interquartile range; NA, not analyzed.*

### Single Nucleotide Variations in Methylation_H and Methylation_L

**FIGURE 2:**
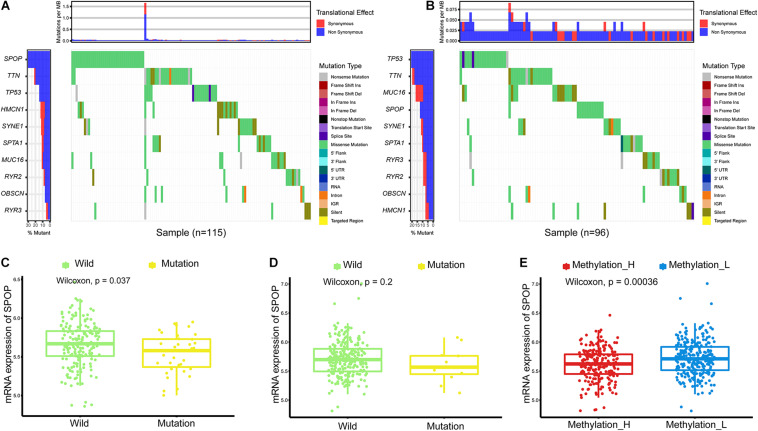
Differences of simple nucleotide variations between Methylation_H and Methylation_L subtypes. **(A)** The map of waterfall for the Methylation_H subtype. **(B)** The map of waterfall for the Methylation_L subtype. **(C)** In the Methylation_H subtype, *SPOP* transcription level of patients with *SPOP* mutation was significantly lower than that of patients with *SPOP* wild-type. **(D)** In the Methylation_L subtype, there was no significant difference in *SPOP* transcription between the mutant and the wild-type patients. **(E)** The differences in the transcription levels of *SPOP* between the Methylation_H and Methylation_L subtypes (PCa, prostate cancer; *SPOP*, speckle-type poz protein. And *p* < 0.05 was defined as statistically significant).

Single nucleotide variations in genes with the top 10 mutation frequencies in these two subtypes are shown in Figures 2A,B. We found that the frequency of single nucleotide variations in *SPOP* was higher in the Methylation_H subtype than in the Methylation_L subtype. *SPOP* is one of the most frequently mutated genes in primary PCa. Based on the tumor-suppressive role of SPOP in PCa and the results of loss-of-function assays, *SPOP* mutations are expected to include the invasion and proliferation of PCa cells (Barbieri et al., 2012; An et al., 2014). Furthermore, within the Methylation_H subtype, mRNA expression levels of *SPOP* in patients with mutations were significantly lower than those in patients with wild-type *SPOP* (Figure 2C). However, this pattern was not observed in the Methylation_L subtype (Figure 2D). Finally, we found that the mRNA expression level of *SPOP* in the Methylation_H subtype was significantly lower than that in the Methylation_L subtype (Figure 2E). These results supported the risk characteristics of Methylation_H.

### Copy Number Alterations of RND3, TMPRSS2–ERG Fusion, and Androgen Receptor Scores Between the Methylation_H and Methylation_L Subtypes

**FIGURE 3:**
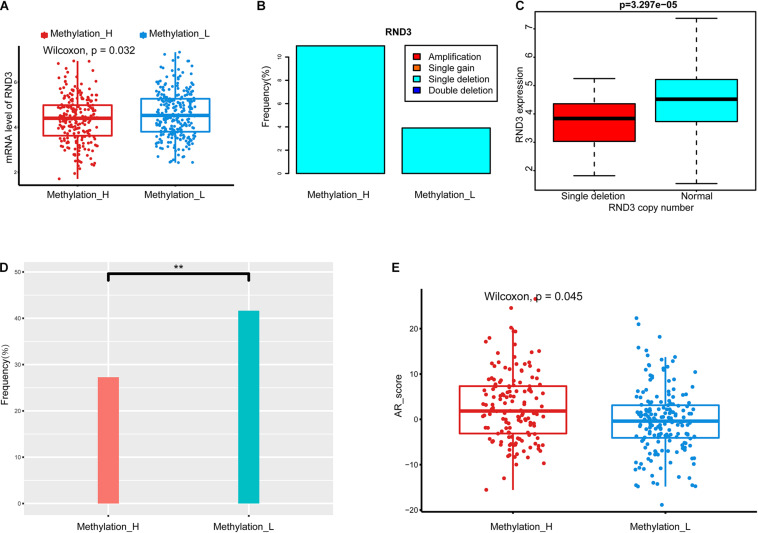
Copy number alterations, TMPRSS2-ERG fusion, and AR scores in each subtype. **(A)** *RND3* had a lower expression level in Methylation_H subtype. **(B)** The frequency of CNA in *RND3* in Methylation_H subtype was significantly higher than that in Methylation_L subtype. **(C)** The expression level of *RND3* was significantly correlated with its CNA, and the expression level of *RND3* was decreased with single deletion. **(D)** Patients in the Methylation_H subtype had a lower frequency of *TMPRSS2-ERG* fusion. **(E)** Patients in the Methylation_H subtype had higher AR scores (CAN, copy number alteration; *RND3*, rho family gtpase 3; AR, androgen receptor. And *p* < 0.05 was defined as statistically significant).

We identified a group of genes with a significant difference in copy number between subtypes. Among these genes, *RND3*, also called *RhoE*, is a tumor suppressor that is downregulated early in the development of PCa (Bektic et al., 2005). Interestingly, the expression of *RND3* was significantly lower in the Methylation_H subtype than that in the Methylation_L subtype (Figure 3A). For *RND3*, the type of CNA and the frequency of CNAs are summarized in Figure 3B. In 477 patients, a single-copy deletion was the only CNA detected in *RND3*. This deletion was more frequent in the Methylation_H subtype. Additionally, *RND3* was significantly down-regulated in cases with the single-copy deletion (Figure 3C).

The frequency of the *TMPRSS2–ERG* fusion was significantly lower in the Methylation_H subtype than in the Methylation_L subtype (Figure 3D). Some studies have revealed that the *TMPRSS2–ERG* fusion is related to the invasiveness of PCa and a higher Gleason score (Perner et al., 2006; Mehra et al., 2007; Rajput et al., 2007; Cheville et al., 2008). However, other studies have reported that the *TMPRSS2–ERG* fusion is not related to prognosis in PCa (Yoshimoto et al., 2006; Tu et al., 2007; Darnel et al., 2009). Furthermore, AR scores for patients in the Methylation_H subtype were higher than those of patients in the Methylation_L subtype (Figure 3E).

### Immune Cells in the Tumor Microenvironment in Each Subtype

**FIGURE 4:**
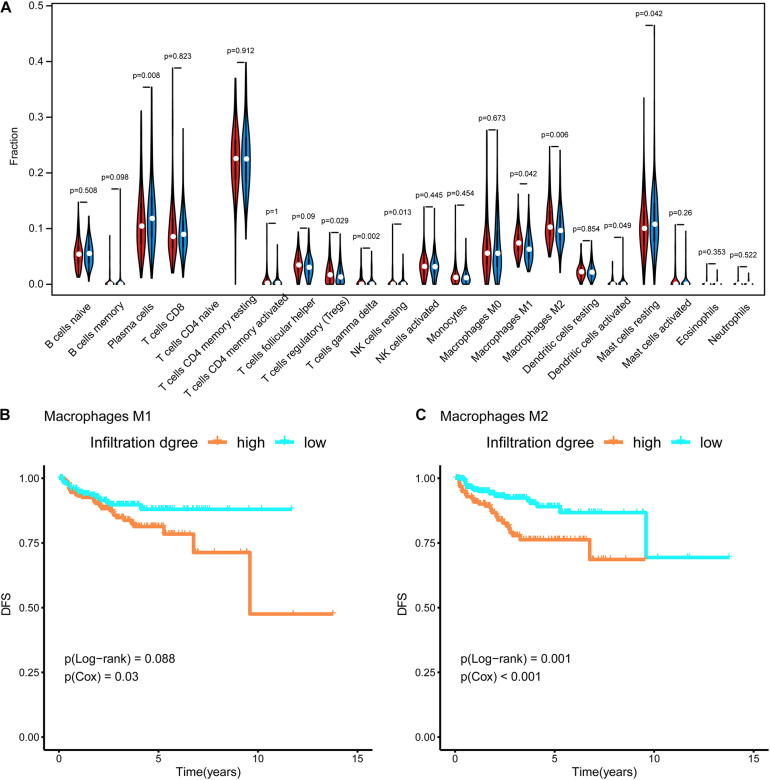
Immune infiltration in each subtype. **(A)** The violin diagram about infiltration degree of 22 kinds of immune cells between Methylation_H and Methylation_L subtypes. **(B)** Survival curves for different levels of Macrophages M1 cells. **(C)** Survival curves for different levels of Macrophages M2 cells (And *p* < 0.05 was defined as statistically significant).

In Figure 4A, the difference in the immune cell composition in the tumor microenvironment of each subtype is displayed in the form of a violin plot. Plasma cells and resting mast cells were significantly less abundant in the Methylation_H subtype and regulatory T cells (Tregs), M1 macrophages, and M2 macrophages were significantly more abundant in the Methylation_H subtype than in the Methylation_L subtype. Among these immune cells, greater M1 and M2 macrophage infiltration in the tumor microenvironment was related to a worse prognosis in PCa (Figures 4B,C).

### Functional Enrichment Analysis of Each Subtype

**FIGURE 5:**
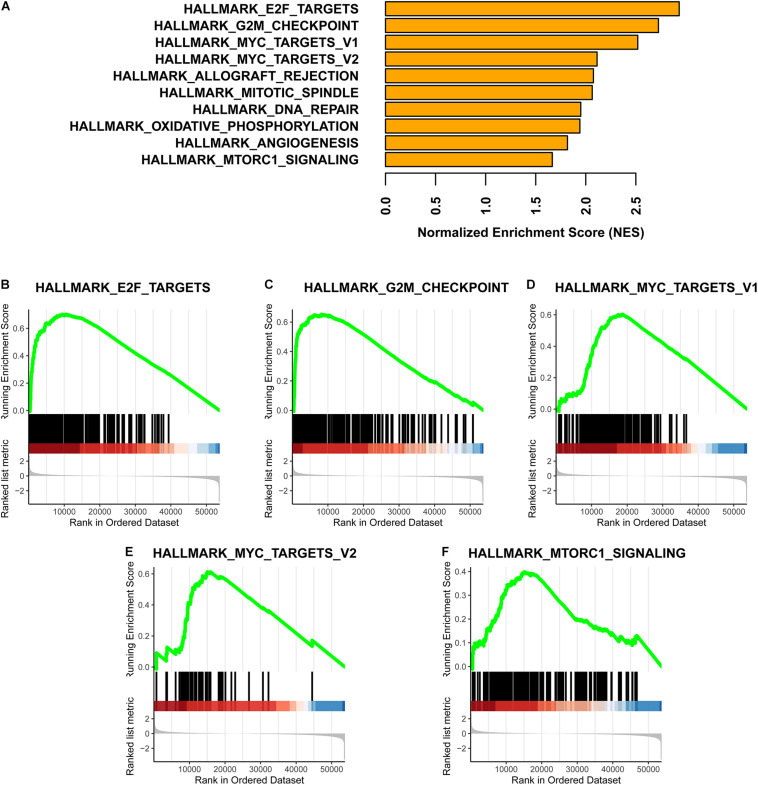
GSEA for these two subtypes. **(A)** Top ten enrichment terms (The enrichment terms were ranked in the descending order of NES). **(B)** HALLMARK_E2F_TARGETS. **(C)** HALLMARK_G2M_CHECKPOINT. **(D)** HALLMARK_MYC_TARGETS_V1. **(E)** HALLMARK_MYC_TARGETS_V2. **(F)** HALLMARK_MTORC1_SIGNALING (NES, normalized enrichment scores. And *p* < 0.05 was defined as statistically significant).

Based on a GSEA, we ranked enriched terms in descending order of normalized enrichment scores. The top ten enriched terms are displayed in the Figure 5A. Among these terms, HALLMARK_E2F_TARGETS, HALLMARK_G2M_ CHECKPOINT, HALLMARK_MYC_TARGETS_V1, HALLMARK_MYC_TARGETS_V2, and HALLMARK_ MTORC1_SIGNALING were enriched in the Methylation_H subtype (Figures 5B–F).

**FIGURE 6:**
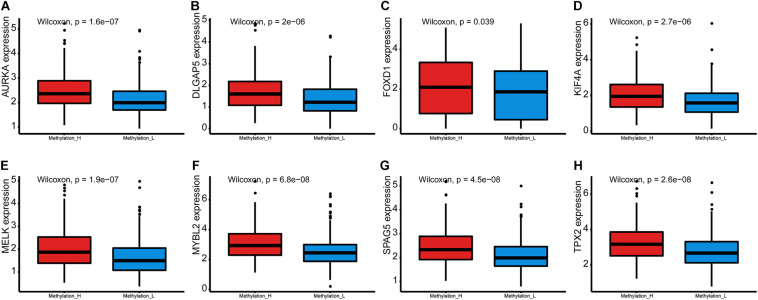
Differences of genes contributing to PCa between Methylation_H and Methylation_L subtypes. **(A)** The mRNA expression levels of *AURKA*. **(B)** The mRNA expression levels of *DLGAP5*. **(C)** The mRNA expression levels of *FOXD1*. **(D)** The mRNA expression levels of *KIF4A*. **(E)** The mRNA expression levels of *MELK*. **(F)** The mRNA expression levels of *MYBL2*. **(G)** The mRNA expression levels of *SPAG5*. **(H)** The mRNA expression levels of and *TPX2* (PCa, prostate cancer. And *p* < 0.05 was defined as statistically significant).

Furthermore, the mRNA expression levels of *AURKA*, *DLGAP5*, *FOXD1*, *KIF4A*, *MELK*, *MYBL2*, *SPAG5*, and *TPX2* were significantly higher in the Methylation_H subtype than in the Methylation_L subtype (Figure 6). These genes have all been reported to facilitate the development and progression of PCa (Kuner et al., 2013; Zhang et al., 2016, 2020b; Akamatsu et al., 2018; Hewit et al., 2018; Zou et al., 2018; Cao et al., 2020; Li et al., 2020).

### WGCNA for the Identification of a Key Gene Module

**FIGURE 7:**
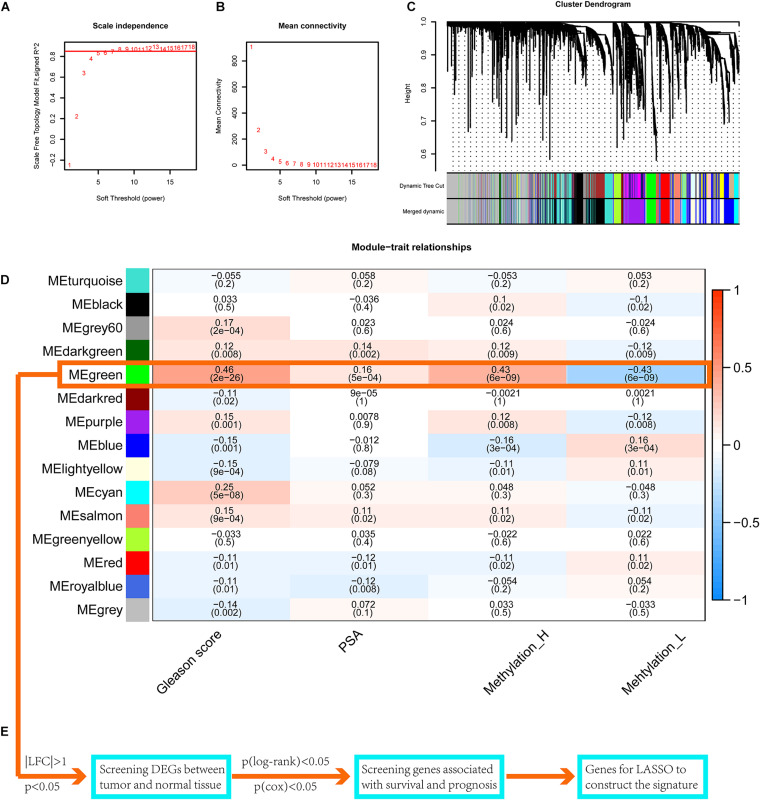
WGCNA to find the genes for the construction of the signature. **(A)** The relationship of soft threshold and TOM-based dissimilarity. **(B)** The relationship of soft threshold and mean connectivity. **(C)** After the dynamic of cut and merged, a total of 15 gene modules were finally generated. **(D)** Heat map for the correlation of gene modules and phenotypes. **(E)** The flow of selection of genes for the signature (WGCNA, weighted correlation network analysis; TOM, topological overlap matrix; DEGs, differentially expressed genes; LASSO, least absolute shrinkage and selection operator. And *p* < 0.05 was defined as statistically significant).

Setting eight as the soft threshold, the independence of the scale-free topology and the mean connectivity in each module were sufficient (Figures 7A,B). After the dynamic cut and merge process, 15 gene modules were generated (Figure 7C). Among these, the MEgreen module was positively correlated with the PSA level, Gleason score, and the Methylation_H subtype (Figure 7D) and was negatively correlated with the Methylation_L subtype. Therefore, genes in this module were related to the development and progression of PCa. According to the flow diagram in Figure 7E, we then screened out the DEGs between PCa and normal prostate tissues in the MEgreen module. Survival-associated DEGs were further identified for LASSO regression.

### Construction of the Gene-Based Risk Signature

**FIGURE 8:**
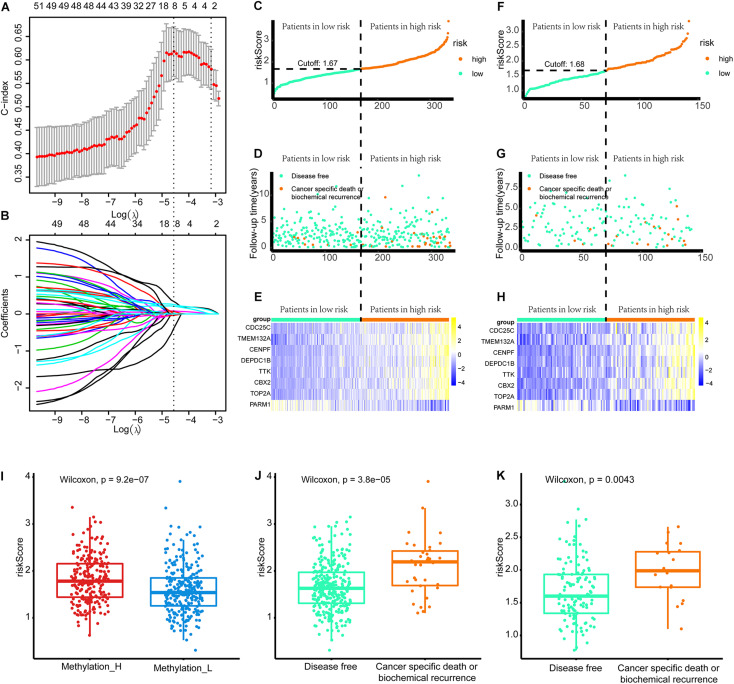
Build the signature by LASSO. **(A)** Cross validation based on C-index to determine the best choice of genes in the signature. **(B)** Genes in the different signatures and their corresponding coefficients. **(C–E)** Patients of training set were arranged in the same ascending order of the risk score. **(F–H)** Patients of internal validation set were arranged in the same ascending order of the risk score. **(C,F)** Patients were divided into different risk levels according to the median of the risk scores in their respective data sets. **(D,G)** The relationship between the survival outcome and risk levels of patients. Low-risk patients were shown on the left side of the dotted line and high-risk patients were shown on the right side. **(E,H)** Heat maps for the genes in the signature. **(I)** Differences in risk scores between the two subtypes. **(J)** Patients with cancer-specific death or biochemical recurrence got higher risk scores in the training set. **(K)** Patients with cancer-specific death or biochemical recurrence got higher risk scores in the internal validation set (LASSO, least absolute shrinkage and selection operator. And *p* < 0.05 was defined as statistically significant).

As shown in Figure 8A, when eight genes were included in the signature, the C-index value was maximized. Accordingly, the eight-gene signature had the best predictive value during the training process. Figure 8B presents the coefficients for each gene during the training process. Finally, an eight-gene signature for predicting the risk score was constructed as follows:

$$\begin{array}{rcl} \mathrm{Risk}\;\mathrm{score} & = & 0.210\times \mathrm{expression}\;\mathrm{of}\;\mathrm{TMEM}132A+0.339\\ & & \times \;\mathrm{expression}\;\mathrm{of}\;\mathrm{CENPF}+0.020\times \mathrm{expression}\;\mathrm{of}\\ & & \mathrm{DEPDC}1B+0.081\times \mathrm{expression}\;\mathrm{of}\;\mathrm{TTK}+0.258\\ & & \times \mathrm{expression}\;\mathrm{of}\;\mathrm{CBX}2+0.065\times \mathrm{expression}\;\mathrm{of}\\ & & \mathrm{TOP}2A-0.388\times \mathrm{expression}\;\mathrm{of}\;\mathrm{CDC}25C-0.038\\ & & \times \mathrm{expression}\;\mathrm{of}\;\mathrm{PARM}1 \end{array}$$

Patients in the training and internal validation set were arranged in ascending order based on risk scores (Figures 8C,F). Setting the median risk score as the threshold, the frequencies of cancer-specific death or biochemical recurrence were higher in high-risk patients than in low-risk patients in the training and internal validation sets (Figures 8D,G). The expression modes of eight genes in the signature are displayed in Figures 8E,H. Furthermore, patients in the Methylation_H subtype had significantly higher risk scores than patients in the Methylation_L subtype (Figure 8I). Finally, we found that patients with cancer-specific death or biochemical recurrence had higher risk scores in the training set, internal validation set, and external validation sets (DKFZ2018, GSE70769, GSE116918, and MSKCC2010) (Figures 8G,K and Supplementary Figure 2).

### Validation of the Signature

**FIGURE 9:**
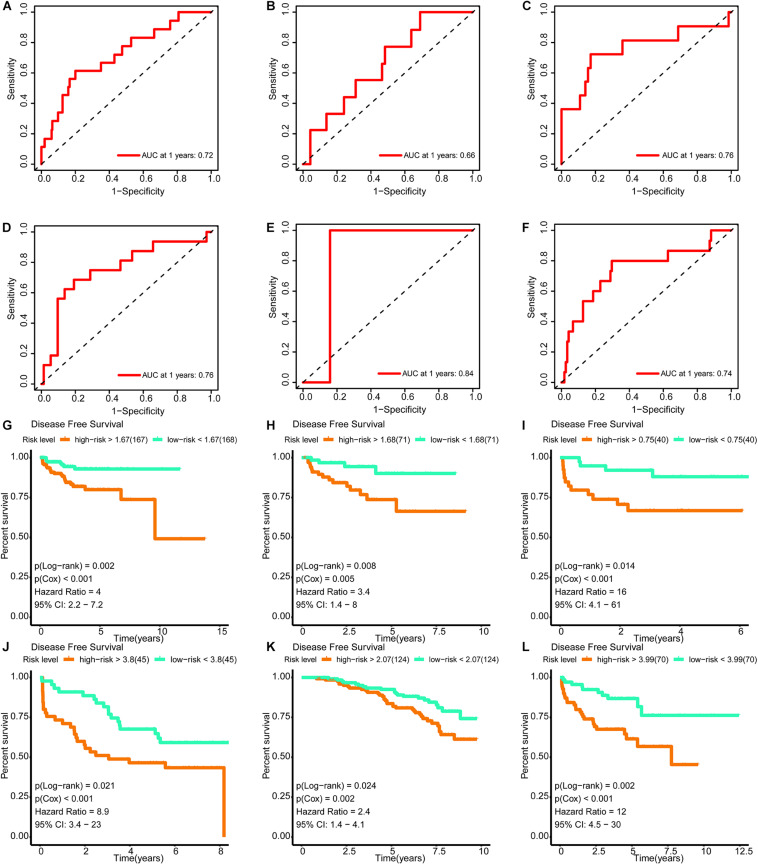
Verification of the effectiveness of the signature. **(A–F)** The ROC curve of 1-year follow-up time. **(G–L)** Kaplan–Meier curve for survival analysis. **(A,G)** The results in the training set. **(B,H)** The results in the internal validation set. **(C,I**) The results in DKFZ2018. **(D,J)** The results in GSE70769. **(E,K)** The results in GSE116918. **(F,L)** The results in MSKCC2010 (AUC, area under curve; DFS, disease-free survival; BCR, biochemical recurrence free survival. And *p* < 0.05 was defined as statistically significant).

The areas under the curve of the tdROC (reflecting the effectiveness of a classifier) for the training set, internal validation set, and external validation sets (DKFZ2018, GSE70769, GSE116918, and MSKCC2010) were 0.72, 0.66, 0.76, 0.76, 0.84, and 0.74, respectively (Figures 9A–F). Furthermore, a survival analysis revealed that a higher risk score was associated with a worse prognosis. Similar results were observed for all data sets evaluated (Figures 9G–L).

**FIGURE 10:**
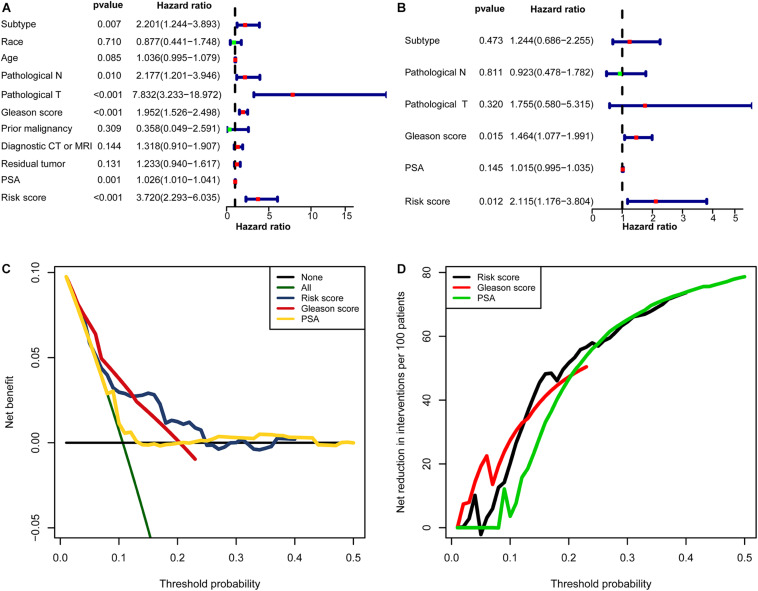
The clinical value of the signature. **(A)** The forest map for univariate COX regression. **(B)** The forest map for multivariate COX regression. **(C,D)** Decision curve analyses suggested that the signature had good clinical benefits. **(C)** The model had higher net benefit and wider threshold probability range. The green line is the net benefit of providing all patients with intervention, and the horizontal black line is the net benefit of providing no patients with intervention. **(D)** The net reduction analyses demonstrated in how many patients an intervention could be avoided without missing any poor prognosis within the effective threshold probability range in **(C)**.

In univariate Cox regression analyses, six variables (subtype, pathological N, pathological T, Gleason score, PSA, and risk score) were associated with a worse prognosis (Figure 10A). In a multivariate Cox regression, Gleason score and risk score were identified as independent indicators for prognosis (Figure 10B). Within a wider range of threshold probabilities, the clinical net benefit was greater for the risk score than for the PSA level or Gleason score (Figure 10C). Within this range of threshold probabilities, the signature provides a more accurate prediction, thereby reducing the number of patients with a worse prognosis (Figure 10D).

## Discussion

We have recently described the advantages and necessity of multi-omics approaches for studies of PCa (Zhang et al., 2020c). In this study, we identified two subtypes with different DNA methylation statuses and found that the Methylation_H subtype was related to a worse prognosis. The subtypes were then comprehensively compared with respect to the epigenome, genome, transcriptome, disease status, immune cell infiltration, and function.

The mRNA levels of *SPOP*, which is the most frequently mutated tumor-suppressor gene in primary PCa, were lower in the Methylation_H subtype than in the Methylation_L subtype (Barbieri et al., 2012; An et al., 2014). Additionally, the mutation frequency in *SPOP* was higher in the Methylation_H subtype, and *SPOP* expression was lower in mutants. Another tumor suppressor that is downregulated early in the development of PCa, *RND3*, was expressed at significantly lower levels in the Methylation_H subtype than in the Methylation_H subtype (Bektic et al., 2005). The single-copy deletion of *RND3* was more frequent in the Methylation_H subtype and this deletion corresponded with the downregulation of *RND3*. AR scores, which reflect disease progression, were also significantly higher in the Methylation_H subtype than in the Methylation_L subtype. M1 and M2 macrophages showed a greater degree of infiltration in Methylation_H. The polarization of macrophages into the M1 and M2 phenotypes plays a pivotal role in ovarian cancer initiation, progression, and metastasis and provides targets for macrophage-centered treatment in the cancer microenvironment (Cheng et al., 2019). Consistent with these previous findings, we found that increased levels of M1 and M2 macrophages in the tumor microenvironment were related to a worse prognosis in PCa.

In the Methylation_H subtype, E2F, MYC, mTORC1, and G2M checkpoint were activated. E2F, MYC, and mTORC1 have been shown to promote the development of PCa (Huang et al., 2017; Zhang et al., 2017; Labbé et al., 2019). Furthermore, G2M checkpoint activation is related to a reduced cancer sensitivity to chemotherapy or radiation (Morgan, 2007). Furthermore, the mRNA expression levels of *AURKA*, *DLGAP5*, *FOXD1*, *KIF4A*, *MELK*, *MYBL2*, *SPAG5*, and *TPX2* were significantly higher in the Methylation_H subtype than in the Methylation_L subtype. The gain of the *AURKA* oncogene is an important genomic change related to treatment-related neuroendocrine PCa (Akamatsu et al., 2018). Androgen-dependent PCa cells need *DLGAP5* to stabilize mitotic health and function, and the knockdown of *DLGAP5* improves the efficacy of docetaxel (Hewit et al., 2018). The knockdown of *FOXD1* and *MYBL2* would inhibit the growth of androgen-independent PCa cells (Li et al., 2020; Zhang et al., 2020b). *KIF4A* plays an significant role in the progression of castration-resistant PCa and serves as a key determinant of resistance to endocrine therapy (Cao et al., 2020). *MELK* is associated with the cell survival rate and BCR in PCa (Jurmeister et al., 2018). *SPAG5* expression is significantly associated with the clinical stage, lymph node metastasis, Gleason score, and BCR (Zhang et al., 2016). The knockdown of *TPX2* increases chromosome mis-segregation and suppresses tumor cell growth in PCa (Pan et al., 2017). These genes driving the progression of PCa were all expressed more highly in the Methylation_H subtype than in the Methylation_L subtype, further supporting the high-risk characteristics of the Methylation_H subtype.

The conserved differences uncovered the high-risk characteristics of the Methylation_H subtype. We further employed WGCNA, a common method in systems biology, to identify a key gene module; this module was related to the Gleason score, PSA, and Methylation_H subtype. Survival-associated DEGs from this gene module were used to construct an eight-gene signature for predicting risk. The effectiveness of the signature was validated in TCGA and another four public datasets (DKFZ2018, GSE70769, GSE116918, and MSKCC2010). With respect to the clinical applications of these findings, we have the following suggestions. Because RNA-seq data in TPM format were used to train the signature, we suggest employing the same data format of data in clinical applications. Considering batch effects of measurement techniques, gene expression levels should be measured by similar techniques, even though the signature performed well in the validation data sets, in which genes were profiled by array-based methods. Furthermore, the risk levels were determined by the median risk score in the patient cohorts. In the future, the study cohort should be further expanded to obtain a more objective and stable threshold range.

Collectively, we identified two subtypes with different methylation statuses at eight CpG sites and evaluated the high-risk characteristics of the Methylation_H subtype based on epigenomics, genomics, transcriptomics, disease status, immune cell infiltration, and functional analyses. Finally, based on these two novel subtypes, an eight-gene predictive signature was constructed and validated using various public datasets.

## Data Availability Statement

The original contributions presented in the study are included in the article/Supplementary Material, further inquiries can be directed to the corresponding author/s.

## Author Contributions

EZ, MZ, and YS were responsible for the design and conception of the research project. EZ, YS, MZ, FS, OM, JH, YG, and HW contributed to the data acquisition or data analysis and data cleaning. EZ and YS participated in the drafting of the manuscript and the rigorous modification of the manuscript to clearly convey the research contents. All authors are responsible for the authenticity and reliability of this study and have no objection to the final submitted manuscript.

## Conflict of Interest

The authors declare that the research was conducted in the absence of any commercial or financial relationships that could be construed as a potential conflict of interest.

## Footnotes

1 https://gco.iarc.fr/

2 https://portal.gdc.cancer.gov/

3 http://asia.ensembl.org/index.html

4 https://www.ncbi.nlm.nih.gov/geo/

5 https://www.cbioportal.org

6 https://cibersortx.stanford.edu/

7 https://www.gsea-msigdb.org/gsea/msigdb/index.jsp
