# Supplementary material for: Establishment of Novel DNA Methylation-Based Prostate Cancer Subtypes and a Risk-Predicting Eight-Gene Signature
Source: Front Cell Dev Biol. 2021 Feb 23;9:639615. doi: 10.3389/fcell.2021.639615 (PMC7940376; doi:10.3389/fcell.2021.639615)
Supplement: Supplementary file 1 [file Table_1.docx]

Supplementary Material

# Supplementary Figure 1.

**(A-C)** For different k values, the figure reflectes the item-consensus of each patient in different clusters. The item-consensus reflects the degree of representation of an individual to different clusters. The greater the value, the more representative the individual is of the characteristics of the corresponding cluster. **(D)** The x-coordinate of the graph is items, and the y-coordinate is the value of k. Each color corresponds to different cluster classification colors in the consensus cluster. If items always change the type of cluster (i.e. change the color in a column) it indicates an unstable classification relationship. If a cluster has a large number of samples with unstable classification, it indicates that the cluster is not a stable classification and cannot become a subtyping. **(E)** The diagram shows the cluster-consensus for each cluster with different k values. The cluster-consensus refers to the average value of the consensus matrix of each cluster, and represents the degree of consensus of this cluster. The higher the cluster-consensus of the cluster, the higher the stability of this cluster.


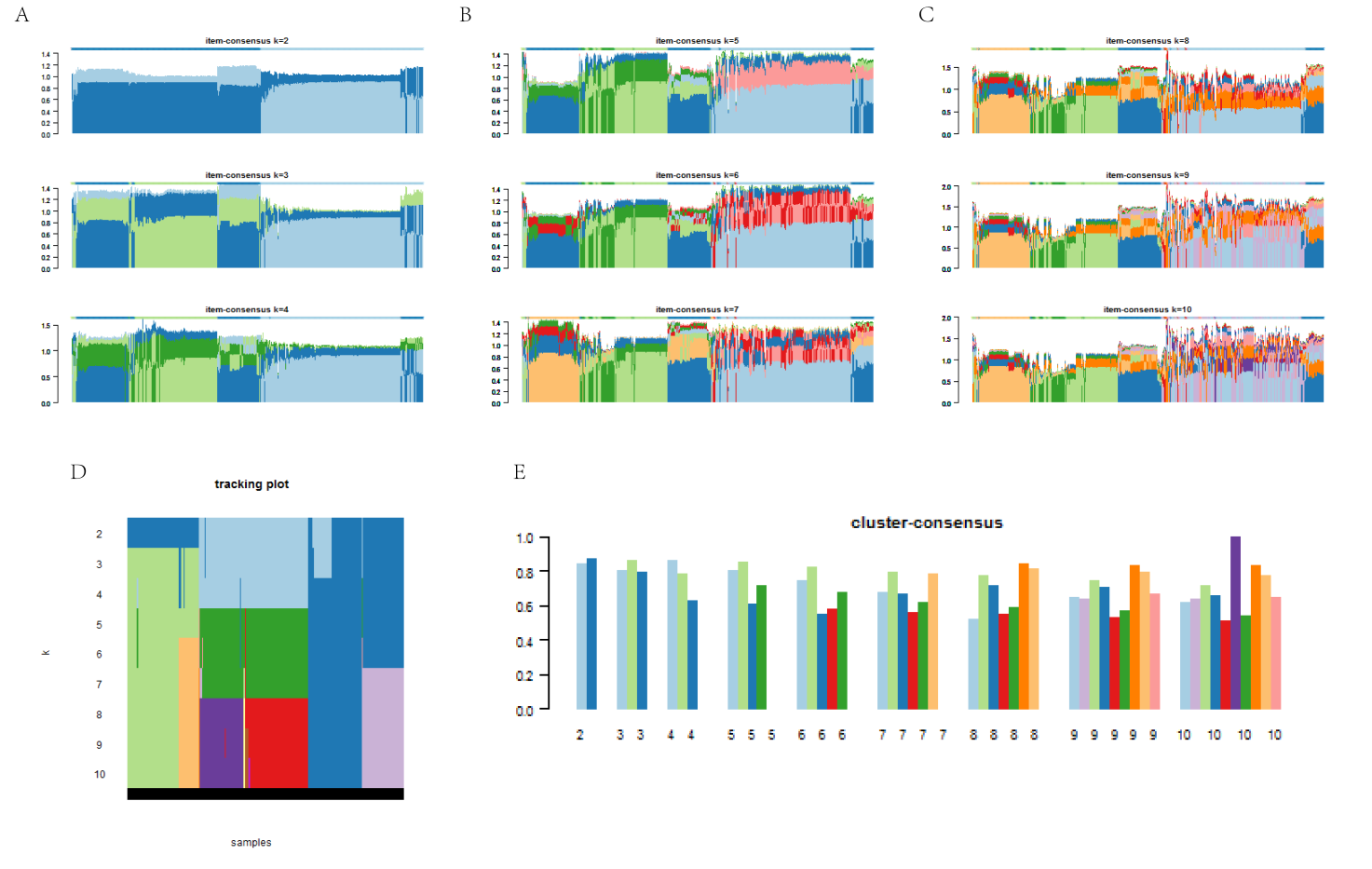


# Supplementary Figure 2

**(A)** Patients with cancer-specific death or biochemical recurrence got higher risk scores in DKFZ2018. **(B)** Patients with cancer-specific death or biochemical recurrence got higher risk scores in GSE70769. **(C)** Patients with cancer-specific death or biochemical recurrence got higher risk scores in GSE116918. **(D)** Patients with cancer-specific death or biochemical recurrence got higher risk scores in MSKCC2010. (And p < 0.05 was defined as statistically significant.)


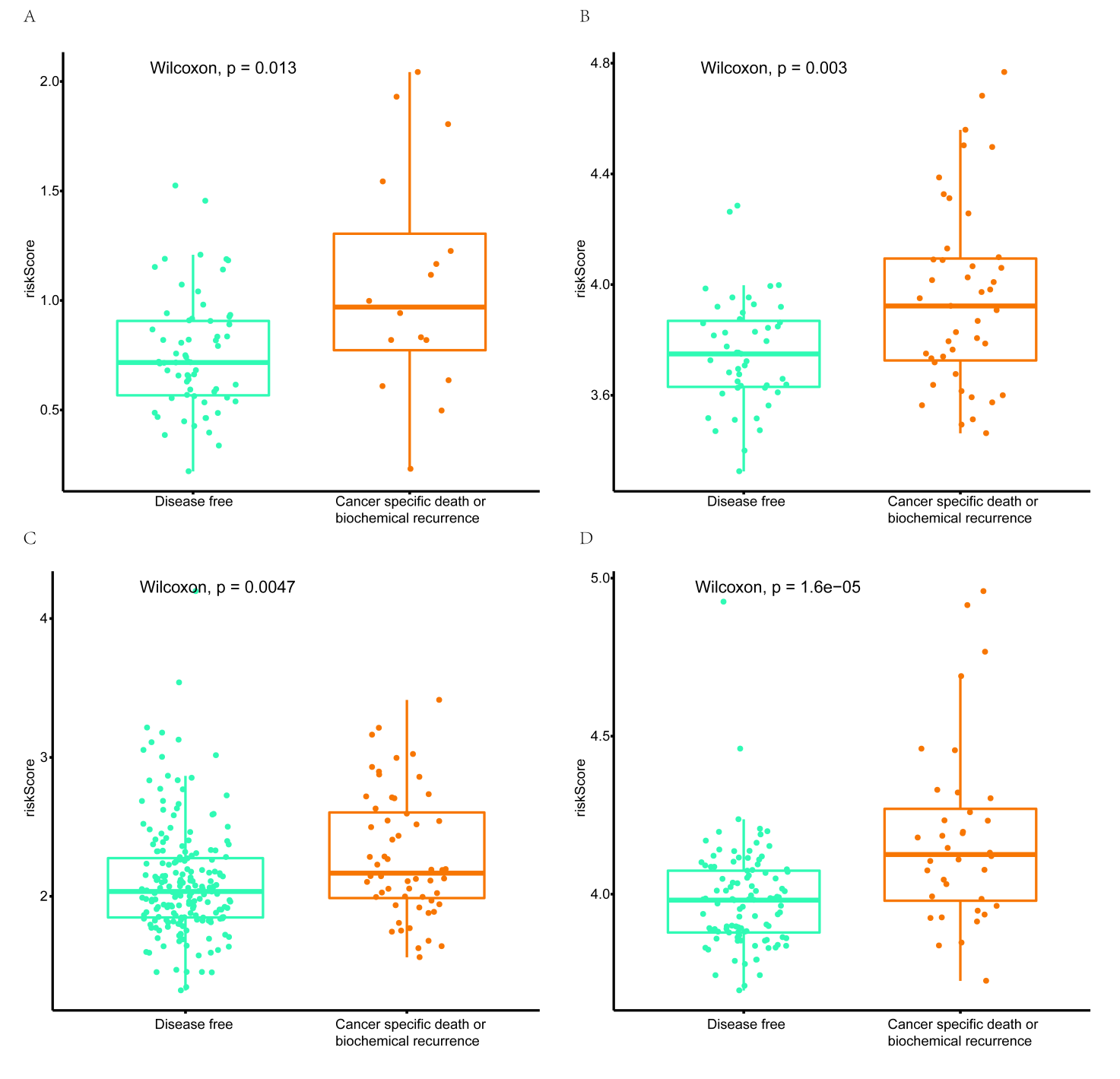


# Supplementary Table 1.

| Supplementary Table 1: Patients in the training set. |
| --- |
| TCGA-HC-7740 |
| TCGA-EJ-A46H |
| TCGA-X4-A8KS |
| TCGA-M7-A720 |
| TCGA-G9-6347 |
| TCGA-EJ-A46E |
| TCGA-FC-A66V |
| TCGA-HC-8258 |
| TCGA-G9-6370 |
| TCGA-KC-A4BR |
| TCGA-CH-5743 |
| TCGA-EJ-A7NJ |
| TCGA-EJ-A46I |
| TCGA-J4-A67O |
| TCGA-EJ-A65G |
| TCGA-M7-A71Y |
| TCGA-EJ-A46B |
| TCGA-YL-A8SK |
| TCGA-VP-A87E |
| TCGA-EJ-AB20 |
| TCGA-G9-6353 |
| TCGA-H9-A6BX |
| TCGA-HI-7169 |
| TCGA-EJ-7786 |
| TCGA-M7-A723 |
| TCGA-KC-A7FE |
| TCGA-CH-5745 |
| TCGA-EJ-5515 |
| TCGA-XK-AAJU |
| TCGA-EJ-A7NG |
| TCGA-EJ-7325 |
| TCGA-XJ-A9DK |
| TCGA-XA-A8JR |
| TCGA-G9-7523 |
| TCGA-VN-A88I |
| TCGA-G9-6385 |
| TCGA-QU-A6IM |
| TCGA-J4-A83L |
| TCGA-J4-AATV |
| TCGA-XJ-A9DQ |
| TCGA-EJ-7328 |
| TCGA-VP-A879 |
| TCGA-EJ-5498 |
| TCGA-FC-7708 |
| TCGA-FC-A8O0 |
| TCGA-G9-7525 |
| TCGA-G9-6333 |
| TCGA-CH-5767 |
| TCGA-ZG-A9L6 |
| TCGA-CH-5789 |
| TCGA-TP-A8TT |
| TCGA-HC-A6AS |
| TCGA-KC-A4BN |
| TCGA-G9-6498 |
| TCGA-G9-6354 |
| TCGA-J4-8200 |
| TCGA-V1-A8WN |
| TCGA-ZG-A8QZ |
| TCGA-EJ-5506 |
| TCGA-2A-AAYF |
| TCGA-KK-A8IL |
| TCGA-EJ-A8FS |
| TCGA-Y6-A8TL |
| TCGA-HC-7745 |
| TCGA-EJ-5503 |
| TCGA-G9-6378 |
| TCGA-KK-A7B2 |
| TCGA-TK-A8OK |
| TCGA-ZG-A8QX |
| TCGA-G9-6343 |
| TCGA-KK-A7AV |
| TCGA-G9-6336 |
| TCGA-KK-A8IG |
| TCGA-HC-7752 |
| TCGA-G9-6351 |
| TCGA-EJ-5502 |
| TCGA-KC-A4BL |
| TCGA-CH-5740 |
| TCGA-EJ-A46D |
| TCGA-J4-A67M |
| TCGA-2A-A8VO |
| TCGA-KK-A8IJ |
| TCGA-HC-7233 |
| TCGA-G9-6499 |
| TCGA-HC-8260 |
| TCGA-QU-A6IO |
| TCGA-HC-7211 |
| TCGA-EJ-8468 |
| TCGA-EJ-A65B |
| TCGA-CH-5771 |
| TCGA-J4-A83M |
| TCGA-HC-8264 |
| TCGA-G9-7522 |
| TCGA-G9-6364 |
| TCGA-J4-AAU2 |
| TCGA-EJ-5526 |
| TCGA-X4-A8KQ |
| TCGA-EJ-A65E |
| TCGA-EJ-5516 |
| TCGA-CH-5762 |
| TCGA-G9-6384 |
| TCGA-EJ-7797 |
| TCGA-HC-7750 |
| TCGA-EJ-A7NH |
| TCGA-EJ-7331 |
| TCGA-J4-A83K |
| TCGA-EJ-A8FU |
| TCGA-G9-6348 |
| TCGA-HC-7079 |
| TCGA-G9-7519 |
| TCGA-HC-7817 |
| TCGA-EJ-7791 |
| TCGA-J4-A67R |
| TCGA-HC-7737 |
| TCGA-G9-6332 |
| TCGA-HC-8259 |
| TCGA-HC-A6AP |
| TCGA-VN-A943 |
| TCGA-EJ-7125 |
| TCGA-G9-7509 |
| TCGA-QU-A6IN |
| TCGA-J4-A83N |
| TCGA-2A-AAYO |
| TCGA-V1-A8ML |
| TCGA-V1-A8X3 |
| TCGA-ZG-A9MC |
| TCGA-G9-6362 |
| TCGA-EJ-7792 |
| TCGA-HC-8256 |
| TCGA-VP-A87C |
| TCGA-EJ-5518 |
| TCGA-G9-6496 |
| TCGA-EJ-5531 |
| TCGA-Y6-A9XI |
| TCGA-J9-A8CP |
| TCGA-QU-A6IL |
| TCGA-VN-A88O |
| TCGA-EJ-7794 |
| TCGA-KK-A7B1 |
| TCGA-HC-A6HX |
| TCGA-HC-7736 |
| TCGA-HC-A9TE |
| TCGA-ZG-A9LU |
| TCGA-HC-A8D0 |
| TCGA-EJ-7317 |
| TCGA-HC-8265 |
| TCGA-KK-A8I9 |
| TCGA-EJ-7318 |
| TCGA-V1-A8MG |
| TCGA-CH-5748 |
| TCGA-KC-A7F3 |
| TCGA-HC-7210 |
| TCGA-EJ-A7NF |
| TCGA-CH-5766 |
| TCGA-YL-A8SR |
| TCGA-CH-5792 |
| TCGA-HC-A6AN |
| TCGA-VN-A88K |
| TCGA-2A-A8W1 |
| TCGA-YL-A8SA |
| TCGA-KK-A8IC |
| TCGA-ZG-A9LS |
| TCGA-EJ-5508 |
| TCGA-ZG-A9LM |
| TCGA-WW-A8ZI |
| TCGA-V1-A9ZK |
| TCGA-ZG-A8QY |
| TCGA-HC-A6AL |
| TCGA-V1-A9Z8 |
| TCGA-J9-A8CN |
| TCGA-EJ-A7NN |
| TCGA-H9-A6BY |
| TCGA-XJ-A83H |
| TCGA-G9-6366 |
| TCGA-EJ-8474 |
| TCGA-MG-AAMC |
| TCGA-FC-A6HD |
| TCGA-G9-6494 |
| TCGA-XK-AAJP |
| TCGA-KK-A7B0 |
| TCGA-EJ-7218 |
| TCGA-KK-A6E6 |
| TCGA-EJ-7781 |
| TCGA-ZG-A8QW |
| TCGA-VN-A88M |
| TCGA-EJ-5521 |
| TCGA-J4-A6G1 |
| TCGA-EJ-5519 |
| TCGA-G9-6329 |
| TCGA-CH-5744 |
| TCGA-YL-A8HL |
| TCGA-J4-A83I |
| TCGA-CH-5737 |
| TCGA-J9-A52B |
| TCGA-CH-5790 |
| TCGA-V1-A9Z9 |
| TCGA-HC-A6HY |
| TCGA-G9-7510 |
| TCGA-EJ-5532 |
| TCGA-J4-A6G3 |
| TCGA-2A-A8VV |
| TCGA-KC-A7FD |
| TCGA-KK-A7AU |
| TCGA-CH-5768 |
| TCGA-HC-7232 |
| TCGA-XJ-A83G |
| TCGA-YL-A8HK |
| TCGA-HC-7819 |
| TCGA-VN-A88L |
| TCGA-XJ-A83F |
| TCGA-SU-A7E7 |
| TCGA-CH-5746 |
| TCGA-KK-A6E4 |
| TCGA-YL-A9WJ |
| TCGA-HC-A8CY |
| TCGA-KK-A8I6 |
| TCGA-HC-A6AO |
| TCGA-YL-A8SC |
| TCGA-J9-A8CK |
| TCGA-HC-7821 |
| TCGA-CH-5764 |
| TCGA-KK-A7AY |
| TCGA-TP-A8TV |
| TCGA-EJ-7115 |
| TCGA-M7-A721 |
| TCGA-J4-A67N |
| TCGA-HC-A76X |
| TCGA-HC-8261 |
| TCGA-V1-A9OA |
| TCGA-VN-A88R |
| TCGA-FC-A5OB |
| TCGA-HC-7080 |
| TCGA-HC-7081 |
| TCGA-HC-7738 |
| TCGA-J4-A6M7 |
| TCGA-EJ-7330 |
| TCGA-G9-6361 |
| TCGA-EJ-7314 |
| TCGA-HC-A76W |
| TCGA-G9-A9S7 |
| TCGA-V1-A9OH |
| TCGA-VN-A88N |
| TCGA-HC-A632 |
| TCGA-EJ-7315 |
| TCGA-KK-A6DY |
| TCGA-CH-5753 |
| TCGA-HC-7749 |
| TCGA-V1-A9OY |
| TCGA-EJ-5522 |
| TCGA-ZG-A9M4 |
| TCGA-2A-A8VT |
| TCGA-EJ-7123 |
| TCGA-VP-A87J |
| TCGA-EJ-5507 |
| TCGA-KC-A7F5 |
| TCGA-XJ-A9DX |
| TCGA-EJ-5494 |
| TCGA-CH-5772 |
| TCGA-EJ-A65J |
| TCGA-KK-A7AQ |
| TCGA-HC-8216 |
| TCGA-M7-A724 |
| TCGA-EJ-5495 |
| TCGA-EJ-5504 |
| TCGA-KK-A6E7 |
| TCGA-V1-A9ZG |
| TCGA-YL-A9WK |
| TCGA-EJ-5509 |
| TCGA-HC-7077 |
| TCGA-KK-A7B3 |
| TCGA-EJ-5514 |
| TCGA-EJ-7788 |
| TCGA-V1-A9OL |
| TCGA-EJ-7783 |
| TCGA-HC-A48F |
| TCGA-KK-A6E2 |
| TCGA-EJ-A65F |
| TCGA-V1-A8WS |
| TCGA-HC-7742 |
| TCGA-KK-A59Y |
| TCGA-HC-7231 |
| TCGA-KK-A8I8 |
| TCGA-KK-A6E0 |
| TCGA-EJ-7327 |
| TCGA-CH-5788 |
| TCGA-G9-A9S4 |
| TCGA-ZG-A9L0 |
| TCGA-HI-7171 |
| TCGA-HI-7168 |
| TCGA-KK-A59X |
| TCGA-CH-5791 |
| TCGA-KK-A8IF |
| TCGA-HC-7212 |
| TCGA-YL-A8SO |
| TCGA-YL-A8S9 |
| TCGA-YL-A8SP |
| TCGA-2A-A8W3 |
| TCGA-VP-A87H |
| TCGA-HC-8266 |
| TCGA-ZG-A9KY |
| TCGA-KK-A5A1 |
| TCGA-YL-A9WH |
| TCGA-YL-A8SL |
| TCGA-YL-A9WL |
| TCGA-J9-A52E |
| TCGA-CH-5769 |
| TCGA-CH-5750 |
| TCGA-YL-A9WI |
| TCGA-M7-A71Z |
| TCGA-ZG-A9LZ |
| TCGA-KK-A59Z |
| TCGA-YL-A8SQ |
| TCGA-V1-A8WW |
| TCGA-CH-5754 |
| TCGA-YL-A8SJ |
| TCGA-YL-A8S8 |
| TCGA-XK-AAIV |
| TCGA-ZG-A9N3 |
| TCGA-ZG-A9L5 |
| TCGA-KK-A8IA |
| TCGA-KK-A59V |
| TCGA-ZG-A9LB |
| TCGA-V1-A8WV |
| TCGA-M7-A725 |
| TCGA-YL-A9WY |
| TCGA-V1-A9ZI |
| TCGA-KK-A8IK |
| TCGA-EJ-7789 |
| TCGA-XK-AAIW |
| TCGA-ZG-A9L1 |
| TCGA-CH-5761 |
| TCGA-CH-5751 |
| TCGA-HC-A4ZV |
| TCGA-EJ-5525 |
| TCGA-V1-A9O5 |

# Supplementary Table 2.

| Supplementary Table 2: Patients in the validation set. |
| --- |
| TCGA-G9-6367 |
| TCGA-J4-A67Q |
| TCGA-EJ-A7NK |
| TCGA-CH-5763 |
| TCGA-HI-7170 |
| TCGA-EJ-A8FN |
| TCGA-2A-A8VL |
| TCGA-V1-A8MU |
| TCGA-VP-A878 |
| TCGA-XK-AAIR |
| TCGA-VN-A88P |
| TCGA-KK-A6E5 |
| TCGA-YL-A9WX |
| TCGA-YL-A8SI |
| TCGA-J4-A67T |
| TCGA-EJ-AB27 |
| TCGA-V1-A8MF |
| TCGA-YL-A8HJ |
| TCGA-G9-6379 |
| TCGA-V1-A8WL |
| TCGA-XK-AAK1 |
| TCGA-J4-A67L |
| TCGA-V1-A9OX |
| TCGA-HC-7820 |
| TCGA-QU-A6IP |
| TCGA-J4-A67S |
| TCGA-KK-A6E3 |
| TCGA-G9-6338 |
| TCGA-HC-7075 |
| TCGA-EJ-7793 |
| TCGA-HC-A6AQ |
| TCGA-V1-A9OQ |
| TCGA-HC-8213 |
| TCGA-CH-5738 |
| TCGA-EJ-A6RC |
| TCGA-HC-8262 |
| TCGA-EJ-5517 |
| TCGA-G9-6339 |
| TCGA-V1-A9OF |
| TCGA-H9-7775 |
| TCGA-YL-A8SB |
| TCGA-G9-6377 |
| TCGA-HC-A8D1 |
| TCGA-XK-AAJT |
| TCGA-EJ-A6RA |
| TCGA-CH-5739 |
| TCGA-HC-7747 |
| TCGA-HC-7209 |
| TCGA-EJ-5511 |
| TCGA-CH-5741 |
| TCGA-EJ-7785 |
| TCGA-EJ-8472 |
| TCGA-EJ-A65M |
| TCGA-EJ-5512 |
| TCGA-YL-A8HO |
| TCGA-EJ-5505 |
| TCGA-HC-7748 |
| TCGA-YL-A8SH |
| TCGA-J9-A52C |
| TCGA-KK-A6E8 |
| TCGA-HC-7818 |
| TCGA-KC-A7FA |
| TCGA-2A-AAYU |
| TCGA-EJ-5530 |
| TCGA-KK-A8I7 |
| TCGA-G9-6365 |
| TCGA-EJ-5527 |
| TCGA-EJ-8470 |
| TCGA-G9-6371 |
| TCGA-G9-6373 |
| TCGA-G9-6342 |
| TCGA-XJ-A9DI |
| TCGA-KK-A7AW |
| TCGA-KK-A8I5 |
| TCGA-G9-6356 |
| TCGA-XK-AAJR |
| TCGA-ZG-A9LN |
| TCGA-ZG-A9LY |
| TCGA-XK-AAJ3 |
| TCGA-M7-A722 |
| TCGA-J4-8198 |
| TCGA-KK-A8IM |
| TCGA-EJ-7321 |
| TCGA-G9-6363 |
| TCGA-HC-8257 |
| TCGA-EJ-5524 |
| TCGA-VP-A876 |
| TCGA-KK-A8I4 |
| TCGA-ZG-A9L9 |
| TCGA-EJ-5510 |
| TCGA-J4-A67K |
| TCGA-G9-6369 |
| TCGA-KC-A7F6 |
| TCGA-XQ-A8TB |
| TCGA-VP-A875 |
| TCGA-EJ-5542 |
| TCGA-EJ-A46F |
| TCGA-2A-A8VX |
| TCGA-ZG-A9ND |
| TCGA-EJ-7312 |
| TCGA-KK-A8IH |
| TCGA-HC-7078 |
| TCGA-EJ-5501 |
| TCGA-VP-A87D |
| TCGA-EJ-7782 |
| TCGA-ZG-A9L2 |
| TCGA-ZG-A9L4 |
| TCGA-J4-A83J |
| TCGA-VN-A88Q |
| TCGA-KK-A7AZ |
| TCGA-V1-A8MM |
| TCGA-XK-AAJA |
| TCGA-HC-A631 |
| TCGA-KK-A7AP |
| TCGA-HC-7230 |
| TCGA-V1-A9O7 |
| TCGA-EJ-5499 |
| TCGA-J4-AATZ |
| TCGA-KK-A8IB |
| TCGA-CH-5765 |
| TCGA-VP-AA1N |
| TCGA-J9-A8CL |
| TCGA-KK-A7B4 |
| TCGA-VP-A87B |
| TCGA-KK-A8ID |
| TCGA-G9-A9S0 |
| TCGA-CH-5794 |
| TCGA-HC-7744 |
| TCGA-KK-A6E1 |
| TCGA-EJ-8469 |
| TCGA-KC-A4BV |
| TCGA-4L-AA1F |
| TCGA-FC-7961 |
| TCGA-EJ-7784 |
| TCGA-ZG-A9NI |
| TCGA-J9-A8CM |
| TCGA-HC-7213 |
| TCGA-CH-5752 |
| TCGA-YL-A8HM |
| TCGA-G9-7521 |
| TCGA-HC-A9TH |
| TCGA-FC-A4JI |

# Supplementary Table 3.

| Supplementary Table 3: The specific subtype grouping of each patient under different k values. | | | | |
| --- | --- | --- | --- | --- |
| TCGA_id | cluster(k=2) | cluster(k=3) | cluster(k=4) | cluster(k=5) |
| TCGA-YL-A9WH | Methylation_H | 1 | 1 | 1 |
| TCGA-HC-7821 | Methylation_H | 1 | 1 | 1 |
| TCGA-M7-A724 | Methylation_H | 1 | 1 | 1 |
| TCGA-M7-A71Z | Methylation_L | 2 | 2 | 2 |
| TCGA-M7-A722 | Methylation_L | 3 | 1 | 1 |
| TCGA-KK-A6E2 | Methylation_H | 1 | 1 | 1 |
| TCGA-HC-8257 | Methylation_L | 3 | 3 | 3 |
| TCGA-HC-7077 | Methylation_H | 1 | 1 | 1 |
| TCGA-G9-6369 | Methylation_H | 1 | 1 | 1 |
| TCGA-EJ-A46F | Methylation_H | 1 | 1 | 1 |
| TCGA-YL-A8SL | Methylation_H | 1 | 1 | 1 |
| TCGA-KK-A59Z | Methylation_H | 1 | 1 | 1 |
| TCGA-EJ-8472 | Methylation_H | 1 | 1 | 1 |
| TCGA-EJ-8474 | Methylation_H | 1 | 1 | 1 |
| TCGA-KK-A8I5 | Methylation_L | 2 | 2 | 4 |
| TCGA-CH-5772 | Methylation_H | 1 | 1 | 1 |
| TCGA-CH-5752 | Methylation_H | 1 | 1 | 1 |
| TCGA-HC-7819 | Methylation_L | 3 | 3 | 3 |
| TCGA-EJ-7312 | Methylation_H | 1 | 3 | 3 |
| TCGA-EJ-7784 | Methylation_L | 2 | 2 | 4 |
| TCGA-G9-6362 | Methylation_H | 1 | 1 | 1 |
| TCGA-H9-7775 | Methylation_L | 2 | 2 | 4 |
| TCGA-HC-A631 | Methylation_H | 1 | 1 | 1 |
| TCGA-HC-7080 | Methylation_H | 1 | 1 | 1 |
| TCGA-KK-A8IA | Methylation_H | 1 | 3 | 3 |
| TCGA-HC-A6AN | Methylation_H | 1 | 1 | 1 |
| TCGA-G9-6332 | Methylation_L | 2 | 2 | 4 |
| TCGA-HC-7231 | Methylation_L | 2 | 2 | 2 |
| TCGA-KK-A6E7 | Methylation_L | 3 | 3 | 3 |
| TCGA-WW-A8ZI | Methylation_L | 2 | 4 | 4 |
| TCGA-KK-A59V | Methylation_H | 1 | 1 | 1 |
| TCGA-YL-A8SQ | Methylation_H | 1 | 1 | 1 |
| TCGA-EJ-5494 | Methylation_L | 3 | 3 | 3 |
| TCGA-G9-7525 | Methylation_L | 2 | 2 | 2 |
| TCGA-ZG-A9KY | Methylation_H | 1 | 1 | 3 |
| TCGA-ZG-A8QY | Methylation_H | 1 | 1 | 1 |
| TCGA-EJ-7314 | Methylation_H | 1 | 3 | 3 |
| TCGA-HC-8261 | Methylation_H | 1 | 1 | 1 |
| TCGA-KK-A7AU | Methylation_H | 1 | 1 | 1 |
| TCGA-EJ-7321 | Methylation_L | 2 | 2 | 2 |
| TCGA-XJ-A83F | Methylation_L | 2 | 2 | 2 |
| TCGA-HC-8213 | Methylation_L | 2 | 2 | 2 |
| TCGA-EJ-A65F | Methylation_H | 1 | 1 | 1 |
| TCGA-J9-A52C | Methylation_H | 1 | 3 | 3 |
| TCGA-XK-AAJP | Methylation_H | 1 | 1 | 1 |
| TCGA-ZG-A9NI | Methylation_H | 1 | 3 | 3 |
| TCGA-CH-5765 | Methylation_H | 1 | 1 | 1 |
| TCGA-YL-A8SH | Methylation_H | 1 | 1 | 1 |
| TCGA-EJ-5526 | Methylation_H | 1 | 1 | 1 |
| TCGA-V1-A8WW | Methylation_L | 2 | 2 | 2 |
| TCGA-KK-A7AW | Methylation_H | 1 | 1 | 1 |
| TCGA-XQ-A8TB | Methylation_H | 1 | 1 | 1 |
| TCGA-FC-A5OB | Methylation_L | 3 | 3 | 3 |
| TCGA-HC-7232 | Methylation_H | 1 | 3 | 3 |
| TCGA-G9-6348 | Methylation_L | 2 | 2 | 4 |
| TCGA-EJ-8468 | Methylation_H | 1 | 1 | 1 |
| TCGA-KK-A7AP | Methylation_L | 2 | 2 | 4 |
| TCGA-HC-8264 | Methylation_L | 2 | 2 | 4 |
| TCGA-HC-8262 | Methylation_L | 2 | 2 | 2 |
| TCGA-CH-5745 | Methylation_H | 1 | 1 | 1 |
| TCGA-HC-7230 | Methylation_L | 2 | 2 | 2 |
| TCGA-KK-A6E6 | Methylation_L | 2 | 2 | 2 |
| TCGA-HC-7233 | Methylation_L | 2 | 2 | 2 |
| TCGA-G9-6361 | Methylation_L | 2 | 2 | 4 |
| TCGA-EJ-7327 | Methylation_H | 1 | 1 | 1 |
| TCGA-G9-7523 | Methylation_H | 1 | 1 | 1 |
| TCGA-EJ-7125 | Methylation_L | 3 | 3 | 3 |
| TCGA-2A-A8VV | Methylation_L | 2 | 2 | 2 |
| TCGA-HC-7078 | Methylation_H | 1 | 1 | 1 |
| TCGA-EJ-7328 | Methylation_H | 1 | 1 | 1 |
| TCGA-G9-A9S4 | Methylation_L | 2 | 2 | 2 |
| TCGA-ZG-A9L5 | Methylation_H | 1 | 1 | 1 |
| TCGA-KK-A7B3 | Methylation_H | 1 | 1 | 1 |
| TCGA-EJ-A7NF | Methylation_L | 2 | 2 | 2 |
| TCGA-EJ-7315 | Methylation_H | 1 | 1 | 1 |
| TCGA-YL-A8S8 | Methylation_H | 1 | 3 | 3 |
| TCGA-G9-6494 | Methylation_L | 2 | 2 | 4 |
| TCGA-CH-5740 | Methylation_L | 2 | 2 | 2 |
| TCGA-KK-A59X | Methylation_H | 1 | 1 | 1 |
| TCGA-VP-A87B | Methylation_L | 2 | 2 | 4 |
| TCGA-VN-A88L | Methylation_L | 2 | 2 | 2 |
| TCGA-HI-7171 | Methylation_H | 1 | 1 | 1 |
| TCGA-HC-7081 | Methylation_H | 1 | 1 | 1 |
| TCGA-V1-A8WS | Methylation_L | 2 | 2 | 4 |
| TCGA-EJ-7115 | Methylation_H | 1 | 1 | 1 |
| TCGA-EJ-5524 | Methylation_L | 2 | 2 | 2 |
| TCGA-EJ-7788 | Methylation_H | 1 | 1 | 5 |
| TCGA-HC-7209 | Methylation_H | 1 | 1 | 1 |
| TCGA-G9-6365 | Methylation_L | 2 | 2 | 2 |
| TCGA-J4-A83N | Methylation_L | 2 | 2 | 4 |
| TCGA-XJ-A9DX | Methylation_H | 1 | 1 | 1 |
| TCGA-H9-A6BY | Methylation_L | 2 | 2 | 2 |
| TCGA-EJ-7325 | Methylation_L | 2 | 2 | 4 |
| TCGA-HC-A76W | Methylation_H | 1 | 1 | 1 |
| TCGA-CH-5791 | Methylation_L | 2 | 2 | 2 |
| TCGA-J9-A8CL | Methylation_H | 1 | 1 | 1 |
| TCGA-HC-7075 | Methylation_L | 3 | 3 | 3 |
| TCGA-EJ-5505 | Methylation_L | 3 | 3 | 3 |
| TCGA-G9-6385 | Methylation_L | 3 | 3 | 3 |
| TCGA-EJ-5527 | Methylation_L | 3 | 3 | 3 |
| TCGA-CH-5739 | Methylation_H | 1 | 1 | 1 |
| TCGA-YL-A9WK | Methylation_L | 2 | 2 | 2 |
| TCGA-QU-A6IN | Methylation_L | 2 | 2 | 4 |
| TCGA-KK-A6E4 | Methylation_H | 1 | 1 | 1 |
| TCGA-CH-5753 | Methylation_H | 1 | 3 | 3 |
| TCGA-HC-8256 | Methylation_H | 1 | 1 | 1 |
| TCGA-G9-6356 | Methylation_L | 2 | 2 | 2 |
| TCGA-KK-A8I9 | Methylation_H | 1 | 1 | 1 |
| TCGA-KC-A7F6 | Methylation_H | 1 | 1 | 1 |
| TCGA-2A-A8VX | Methylation_L | 2 | 2 | 4 |
| TCGA-VP-A87H | Methylation_H | 1 | 1 | 1 |
| TCGA-HC-8266 | Methylation_L | 3 | 3 | 3 |
| TCGA-YL-A8SP | Methylation_L | 2 | 2 | 2 |
| TCGA-HC-7820 | Methylation_L | 2 | 2 | 2 |
| TCGA-KK-A59Y | Methylation_L | 2 | 2 | 2 |
| TCGA-G9-6373 | Methylation_L | 2 | 2 | 2 |
| TCGA-CH-5751 | Methylation_H | 1 | 3 | 3 |
| TCGA-G9-6336 | Methylation_L | 2 | 2 | 2 |
| TCGA-J4-A67K | Methylation_L | 2 | 2 | 2 |
| TCGA-EJ-8469 | Methylation_H | 1 | 1 | 1 |
| TCGA-HC-7749 | Methylation_L | 2 | 2 | 4 |
| TCGA-HC-A76X | Methylation_L | 2 | 2 | 2 |
| TCGA-EJ-7318 | Methylation_H | 1 | 1 | 1 |
| TCGA-V1-A8X3 | Methylation_L | 2 | 2 | 2 |
| TCGA-EJ-8470 | Methylation_H | 1 | 1 | 1 |
| TCGA-KC-A7FD | Methylation_H | 1 | 1 | 1 |
| TCGA-EJ-5508 | Methylation_L | 2 | 2 | 2 |
| TCGA-FC-7708 | Methylation_H | 1 | 1 | 1 |
| TCGA-ZG-A9LB | Methylation_L | 3 | 3 | 3 |
| TCGA-HC-A6AQ | Methylation_H | 1 | 1 | 1 |
| TCGA-G9-6333 | Methylation_H | 1 | 1 | 1 |
| TCGA-J4-A67S | Methylation_H | 1 | 1 | 1 |
| TCGA-YL-A8S9 | Methylation_H | 1 | 1 | 1 |
| TCGA-CH-5764 | Methylation_H | 1 | 1 | 1 |
| TCGA-G9-6377 | Methylation_L | 3 | 3 | 3 |
| TCGA-VP-A875 | Methylation_H | 1 | 1 | 1 |
| TCGA-G9-6371 | Methylation_H | 1 | 1 | 1 |
| TCGA-J4-A67T | Methylation_L | 2 | 2 | 2 |
| TCGA-HC-8259 | Methylation_L | 2 | 2 | 2 |
| TCGA-CH-5768 | Methylation_H | 1 | 1 | 1 |
| TCGA-EJ-7783 | Methylation_H | 1 | 3 | 3 |
| TCGA-CH-5750 | Methylation_L | 2 | 2 | 4 |
| TCGA-CH-5790 | Methylation_H | 1 | 1 | 1 |
| TCGA-YL-A8SR | Methylation_H | 1 | 1 | 1 |
| TCGA-YL-A8SA | Methylation_L | 2 | 2 | 2 |
| TCGA-J4-A83M | Methylation_H | 1 | 1 | 1 |
| TCGA-G9-6351 | Methylation_H | 1 | 1 | 1 |
| TCGA-EJ-7786 | Methylation_L | 3 | 3 | 3 |
| TCGA-2A-A8W3 | Methylation_H | 1 | 1 | 1 |
| TCGA-G9-6339 | Methylation_H | 1 | 3 | 3 |
| TCGA-YL-A8SC | Methylation_H | 1 | 1 | 1 |
| TCGA-HC-7745 | Methylation_L | 3 | 3 | 3 |
| TCGA-EJ-7317 | Methylation_H | 1 | 1 | 1 |
| TCGA-J4-A83I | Methylation_L | 2 | 2 | 2 |
| TCGA-HC-7212 | Methylation_L | 2 | 2 | 2 |
| TCGA-G9-6338 | Methylation_H | 1 | 1 | 1 |
| TCGA-EJ-A65E | Methylation_H | 1 | 1 | 1 |
| TCGA-HC-A8CY | Methylation_H | 1 | 1 | 1 |
| TCGA-Y6-A8TL | Methylation_H | 1 | 1 | 1 |
| TCGA-ZG-A8QZ | Methylation_H | 1 | 3 | 3 |
| TCGA-YL-A8SB | Methylation_L | 2 | 2 | 4 |
| TCGA-VP-A876 | Methylation_L | 2 | 2 | 2 |
| TCGA-2A-A8VO | Methylation_L | 3 | 3 | 3 |
| TCGA-EJ-A6RC | Methylation_L | 3 | 3 | 3 |
| TCGA-VN-A88M | Methylation_L | 2 | 2 | 2 |
| TCGA-V1-A9OF | Methylation_H | 1 | 1 | 1 |
| TCGA-EJ-A8FS | Methylation_L | 2 | 2 | 4 |
| TCGA-V1-A9OA | Methylation_L | 3 | 3 | 3 |
| TCGA-G9-6342 | Methylation_L | 2 | 2 | 4 |
| TCGA-EJ-7331 | Methylation_H | 1 | 1 | 1 |
| TCGA-VP-A87D | Methylation_L | 2 | 2 | 2 |
| TCGA-HC-A6AS | Methylation_H | 1 | 1 | 1 |
| TCGA-G9-6354 | Methylation_L | 2 | 2 | 2 |
| TCGA-J4-A83L | Methylation_L | 3 | 3 | 3 |
| TCGA-J9-A8CK | Methylation_L | 2 | 2 | 2 |
| TCGA-V1-A8ML | Methylation_L | 3 | 3 | 3 |
| TCGA-EJ-A65M | Methylation_H | 1 | 1 | 1 |
| TCGA-HC-7736 | Methylation_H | 1 | 1 | 1 |
| TCGA-EJ-7785 | Methylation_L | 2 | 2 | 2 |
| TCGA-CH-5744 | Methylation_H | 1 | 1 | 1 |
| TCGA-QU-A6IP | Methylation_L | 2 | 2 | 4 |
| TCGA-G9-6363 | Methylation_H | 1 | 1 | 1 |
| TCGA-KK-A7AY | Methylation_H | 1 | 1 | 1 |
| TCGA-HC-8258 | Methylation_L | 3 | 3 | 3 |
| TCGA-HC-7818 | Methylation_H | 1 | 1 | 1 |
| TCGA-KK-A8I8 | Methylation_H | 1 | 1 | 1 |
| TCGA-J4-A67L | Methylation_L | 3 | 3 | 3 |
| TCGA-EJ-5509 | Methylation_H | 1 | 1 | 1 |
| TCGA-G9-6384 | Methylation_H | 1 | 1 | 1 |
| TCGA-G9-6498 | Methylation_L | 3 | 3 | 3 |
| TCGA-HI-7169 | Methylation_L | 2 | 2 | 2 |
| TCGA-J4-A67R | Methylation_H | 1 | 1 | 1 |
| TCGA-KC-A4BV | Methylation_H | 1 | 1 | 1 |
| TCGA-KC-A4BN | Methylation_L | 3 | 3 | 3 |
| TCGA-HC-7750 | Methylation_L | 3 | 3 | 3 |
| TCGA-KK-A7B1 | Methylation_L | 2 | 2 | 2 |
| TCGA-J9-A8CN | Methylation_L | 3 | 3 | 3 |
| TCGA-KK-A8IH | Methylation_L | 3 | 3 | 4 |
| TCGA-J4-A6M7 | Methylation_H | 1 | 1 | 1 |
| TCGA-KK-A8IJ | Methylation_L | 3 | 3 | 3 |
| TCGA-J4-A83K | Methylation_L | 2 | 2 | 2 |
| TCGA-J9-A52B | Methylation_H | 1 | 1 | 1 |
| TCGA-YL-A8HO | Methylation_L | 3 | 3 | 3 |
| TCGA-HI-7170 | Methylation_L | 3 | 3 | 3 |
| TCGA-EJ-7792 | Methylation_L | 3 | 3 | 3 |
| TCGA-YL-A8HJ | Methylation_H | 1 | 1 | 1 |
| TCGA-EJ-5542 | Methylation_L | 2 | 2 | 2 |
| TCGA-V1-A9Z8 | Methylation_L | 2 | 2 | 2 |
| TCGA-EJ-7789 | Methylation_L | 3 | 3 | 3 |
| TCGA-EJ-5522 | Methylation_L | 2 | 2 | 2 |
| TCGA-G9-6364 | Methylation_L | 2 | 2 | 2 |
| TCGA-ZG-A9ND | Methylation_H | 1 | 1 | 1 |
| TCGA-CH-5746 | Methylation_L | 2 | 2 | 2 |
| TCGA-KK-A5A1 | Methylation_H | 1 | 1 | 1 |
| TCGA-V1-A9O5 | Methylation_L | 2 | 2 | 2 |
| TCGA-XK-AAJT | Methylation_H | 1 | 1 | 1 |
| TCGA-EJ-5502 | Methylation_L | 3 | 3 | 3 |
| TCGA-G9-7519 | Methylation_L | 3 | 3 | 3 |
| TCGA-YL-A9WI | Methylation_H | 1 | 1 | 1 |
| TCGA-2A-A8VL | Methylation_L | 3 | 3 | 3 |
| TCGA-2A-AAYF | Methylation_L | 3 | 3 | 3 |
| TCGA-HC-7748 | Methylation_L | 2 | 2 | 4 |
| TCGA-J4-8198 | Methylation_L | 2 | 2 | 4 |
| TCGA-KK-A6E0 | Methylation_H | 1 | 1 | 1 |
| TCGA-HC-7747 | Methylation_L | 2 | 2 | 2 |
| TCGA-FC-A6HD | Methylation_L | 3 | 3 | 3 |
| TCGA-FC-A8O0 | Methylation_L | 3 | 3 | 3 |
| TCGA-G9-7509 | Methylation_L | 2 | 2 | 2 |
| TCGA-TP-A8TT | Methylation_L | 2 | 2 | 2 |
| TCGA-J4-A67N | Methylation_L | 2 | 2 | 2 |
| TCGA-J4-8200 | Methylation_L | 3 | 3 | 3 |
| TCGA-HC-A4ZV | Methylation_H | 1 | 1 | 1 |
| TCGA-EJ-7330 | Methylation_H | 1 | 1 | 1 |
| TCGA-G9-7510 | Methylation_H | 1 | 1 | 1 |
| TCGA-G9-6378 | Methylation_L | 2 | 2 | 2 |
| TCGA-VN-A88O | Methylation_L | 3 | 3 | 3 |
| TCGA-J4-A67M | Methylation_H | 1 | 1 | 1 |
| TCGA-EJ-5532 | Methylation_L | 3 | 3 | 3 |
| TCGA-YL-A8SK | Methylation_L | 3 | 3 | 3 |
| TCGA-HC-7740 | Methylation_L | 3 | 3 | 3 |
| TCGA-EJ-7793 | Methylation_L | 2 | 2 | 2 |
| TCGA-ZG-A8QW | Methylation_H | 1 | 3 | 3 |
| TCGA-CH-5741 | Methylation_L | 2 | 2 | 2 |
| TCGA-HC-7079 | Methylation_L | 3 | 3 | 3 |
| TCGA-KK-A6DY | Methylation_H | 1 | 1 | 1 |
| TCGA-ZG-A8QX | Methylation_H | 1 | 1 | 1 |
| TCGA-J9-A52E | Methylation_L | 2 | 2 | 2 |
| TCGA-SU-A7E7 | Methylation_L | 2 | 2 | 2 |
| TCGA-YL-A9WL | Methylation_H | 1 | 1 | 1 |
| TCGA-J4-A67O | Methylation_H | 1 | 1 | 1 |
| TCGA-CH-5767 | Methylation_H | 1 | 1 | 1 |
| TCGA-XA-A8JR | Methylation_L | 3 | 3 | 3 |
| TCGA-CH-5738 | Methylation_L | 2 | 2 | 2 |
| TCGA-G9-6499 | Methylation_H | 1 | 1 | 1 |
| TCGA-G9-6329 | Methylation_H | 1 | 1 | 1 |
| TCGA-EJ-A65G | Methylation_L | 3 | 3 | 3 |
| TCGA-VP-A87C | Methylation_H | 1 | 1 | 1 |
| TCGA-KK-A8IF | Methylation_L | 3 | 3 | 3 |
| TCGA-J4-A6G3 | Methylation_H | 1 | 1 | 1 |
| TCGA-EJ-5519 | Methylation_L | 2 | 2 | 2 |
| TCGA-EJ-5495 | Methylation_H | 1 | 1 | 1 |
| TCGA-ZG-A9L0 | Methylation_H | 1 | 1 | 1 |
| TCGA-EJ-5504 | Methylation_H | 1 | 1 | 1 |
| TCGA-ZG-A9L6 | Methylation_H | 1 | 3 | 3 |
| TCGA-KK-A7AV | Methylation_H | 1 | 1 | 1 |
| TCGA-EJ-5530 | Methylation_L | 2 | 2 | 2 |
| TCGA-HC-A48F | Methylation_L | 2 | 2 | 2 |
| TCGA-HC-A632 | Methylation_H | 1 | 1 | 1 |
| TCGA-G9-6379 | Methylation_H | 1 | 1 | 1 |
| TCGA-V1-A8WN | Methylation_L | 3 | 3 | 3 |
| TCGA-V1-A8MF | Methylation_L | 3 | 3 | 3 |
| TCGA-EJ-7123 | Methylation_H | 1 | 1 | 1 |
| TCGA-KK-A7B4 | Methylation_H | 1 | 1 | 1 |
| TCGA-ZG-A9LN | Methylation_H | 1 | 3 | 3 |
| TCGA-CH-5748 | Methylation_L | 3 | 3 | 3 |
| TCGA-KK-A8IL | Methylation_H | 1 | 1 | 1 |
| TCGA-G9-6367 | Methylation_L | 3 | 3 | 3 |
| TCGA-2A-A8VT | Methylation_H | 1 | 1 | 1 |
| TCGA-YL-A8HM | Methylation_H | 1 | 1 | 1 |
| TCGA-FC-7961 | Methylation_L | 3 | 3 | 3 |
| TCGA-QU-A6IM | Methylation_H | 1 | 1 | 1 |
| TCGA-V1-A8MM | Methylation_H | 1 | 1 | 1 |
| TCGA-MG-AAMC | Methylation_L | 2 | 2 | 4 |
| TCGA-EJ-A46D | Methylation_L | 3 | 3 | 3 |
| TCGA-VN-A88P | Methylation_H | 1 | 1 | 1 |
| TCGA-G9-6353 | Methylation_L | 3 | 3 | 3 |
| TCGA-VN-A88K | Methylation_L | 2 | 2 | 2 |
| TCGA-TK-A8OK | Methylation_H | 1 | 1 | 1 |
| TCGA-G9-6343 | Methylation_L | 3 | 3 | 3 |
| TCGA-KC-A7FE | Methylation_L | 3 | 3 | 3 |
| TCGA-EJ-5501 | Methylation_L | 2 | 2 | 2 |
| TCGA-X4-A8KQ | Methylation_H | 1 | 1 | 1 |
| TCGA-KK-A7B0 | Methylation_H | 1 | 3 | 3 |
| TCGA-VP-A87E | Methylation_L | 3 | 3 | 3 |
| TCGA-VN-A88Q | Methylation_H | 1 | 1 | 1 |
| TCGA-CH-5762 | Methylation_H | 1 | 3 | 3 |
| TCGA-EJ-5499 | Methylation_H | 1 | 1 | 1 |
| TCGA-CH-5771 | Methylation_L | 3 | 3 | 3 |
| TCGA-CH-5761 | Methylation_H | 1 | 1 | 1 |
| TCGA-TP-A8TV | Methylation_L | 3 | 3 | 3 |
| TCGA-V1-A8MG | Methylation_H | 1 | 1 | 1 |
| TCGA-G9-6366 | Methylation_L | 2 | 2 | 4 |
| TCGA-CH-5794 | Methylation_H | 1 | 1 | 1 |
| TCGA-G9-7522 | Methylation_L | 3 | 3 | 3 |
| TCGA-ZG-A9LM | Methylation_L | 2 | 3 | 3 |
| TCGA-J4-A67Q | Methylation_L | 3 | 3 | 3 |
| TCGA-HC-7752 | Methylation_L | 2 | 2 | 2 |
| TCGA-M7-A721 | Methylation_H | 1 | 1 | 1 |
| TCGA-EJ-5516 | Methylation_H | 1 | 1 | 1 |
| TCGA-V1-A9ZG | Methylation_H | 1 | 1 | 1 |
| TCGA-M7-A723 | Methylation_L | 3 | 3 | 3 |
| TCGA-YL-A8SJ | Methylation_H | 1 | 1 | 1 |
| TCGA-KK-A8I7 | Methylation_L | 3 | 3 | 3 |
| TCGA-EJ-AB20 | Methylation_L | 2 | 2 | 2 |
| TCGA-M7-A725 | Methylation_L | 2 | 2 | 4 |
| TCGA-EJ-5511 | Methylation_L | 2 | 2 | 2 |
| TCGA-XK-AAIW | Methylation_H | 1 | 1 | 1 |
| TCGA-EJ-7791 | Methylation_L | 3 | 3 | 3 |
| TCGA-HC-A6AO | Methylation_H | 1 | 1 | 1 |
| TCGA-ZG-A9L4 | Methylation_H | 1 | 3 | 3 |
| TCGA-XJ-A83H | Methylation_H | 1 | 1 | 1 |
| TCGA-VP-A878 | Methylation_H | 1 | 3 | 3 |
| TCGA-EJ-A46I | Methylation_L | 3 | 3 | 3 |
| TCGA-EJ-5512 | Methylation_L | 3 | 3 | 3 |
| TCGA-V1-A9OY | Methylation_L | 2 | 2 | 2 |
| TCGA-XK-AAIR | Methylation_L | 3 | 3 | 3 |
| TCGA-EJ-5498 | Methylation_L | 3 | 3 | 3 |
| TCGA-J4-AAU2 | Methylation_L | 3 | 3 | 3 |
| TCGA-HC-A6HY | Methylation_L | 2 | 2 | 2 |
| TCGA-EJ-7797 | Methylation_H | 1 | 1 | 1 |
| TCGA-V1-A8WL | Methylation_L | 3 | 3 | 3 |
| TCGA-2A-A8W1 | Methylation_H | 1 | 1 | 1 |
| TCGA-HC-A6AP | Methylation_H | 1 | 1 | 1 |
| TCGA-KC-A4BR | Methylation_L | 3 | 3 | 3 |
| TCGA-KK-A7B2 | Methylation_L | 3 | 3 | 3 |
| TCGA-KK-A8I4 | Methylation_H | 1 | 1 | 1 |
| TCGA-YL-A9WJ | Methylation_L | 3 | 3 | 3 |
| TCGA-KK-A8IM | Methylation_L | 2 | 2 | 2 |
| TCGA-HC-7737 | Methylation_L | 3 | 3 | 3 |
| TCGA-G9-6370 | Methylation_L | 3 | 3 | 3 |
| TCGA-VN-A943 | Methylation_H | 1 | 1 | 1 |
| TCGA-VP-A879 | Methylation_L | 2 | 2 | 2 |
| TCGA-KK-A7AQ | Methylation_H | 1 | 1 | 1 |
| TCGA-ZG-A9L9 | Methylation_L | 2 | 2 | 2 |
| TCGA-HC-7210 | Methylation_L | 3 | 3 | 3 |
| TCGA-J4-A6G1 | Methylation_H | 1 | 1 | 1 |
| TCGA-HC-8216 | Methylation_L | 2 | 2 | 2 |
| TCGA-CH-5792 | Methylation_H | 1 | 3 | 3 |
| TCGA-KK-A8ID | Methylation_H | 1 | 1 | 1 |
| TCGA-EJ-7781 | Methylation_L | 3 | 3 | 3 |
| TCGA-EJ-5517 | Methylation_H | 1 | 1 | 1 |
| TCGA-G9-6347 | Methylation_L | 3 | 3 | 3 |
| TCGA-ZG-A9L2 | Methylation_H | 1 | 3 | 3 |
| TCGA-ZG-A9LZ | Methylation_H | 1 | 1 | 1 |
| TCGA-HC-A8D1 | Methylation_L | 3 | 3 | 3 |
| TCGA-QU-A6IO | Methylation_H | 1 | 1 | 1 |
| TCGA-HC-A6AL | Methylation_H | 1 | 1 | 1 |
| TCGA-VP-A87J | Methylation_H | 1 | 1 | 1 |
| TCGA-CH-5769 | Methylation_H | 1 | 1 | 1 |
| TCGA-EJ-7794 | Methylation_L | 3 | 3 | 3 |
| TCGA-M7-A720 | Methylation_L | 3 | 3 | 3 |
| TCGA-EJ-5518 | Methylation_H | 1 | 1 | 1 |
| TCGA-V1-A9OQ | Methylation_H | 1 | 1 | 1 |
| TCGA-KK-A6E5 | Methylation_H | 1 | 1 | 1 |
| TCGA-EJ-5510 | Methylation_H | 1 | 1 | 1 |
| TCGA-QU-A6IL | Methylation_L | 3 | 3 | 3 |
| TCGA-VN-A88I | Methylation_L | 3 | 3 | 3 |
| TCGA-VN-A88N | Methylation_L | 3 | 3 | 3 |
| TCGA-HC-8260 | Methylation_H | 1 | 1 | 1 |
| TCGA-2A-AAYO | Methylation_L | 2 | 2 | 4 |
| TCGA-YL-A8SO | Methylation_H | 1 | 1 | 1 |
| TCGA-G9-6496 | Methylation_L | 3 | 3 | 4 |
| TCGA-CH-5737 | Methylation_L | 3 | 3 | 3 |
| TCGA-CH-5763 | Methylation_L | 3 | 3 | 3 |
| TCGA-EJ-5525 | Methylation_L | 2 | 2 | 2 |
| TCGA-HI-7168 | Methylation_H | 1 | 1 | 1 |
| TCGA-EJ-AB27 | Methylation_L | 3 | 3 | 3 |
| TCGA-KK-A6E3 | Methylation_L | 3 | 3 | 3 |
| TCGA-EJ-A46B | Methylation_L | 3 | 3 | 3 |
| TCGA-HC-7742 | Methylation_L | 2 | 2 | 2 |
| TCGA-EJ-5507 | Methylation_L | 3 | 3 | 3 |
| TCGA-KK-A7AZ | Methylation_L | 2 | 2 | 2 |
| TCGA-G9-A9S0 | Methylation_H | 1 | 1 | 1 |
| TCGA-EJ-A6RA | Methylation_H | 1 | 3 | 3 |
| TCGA-YL-A8SI | Methylation_H | 1 | 1 | 1 |
| TCGA-HC-7211 | Methylation_L | 3 | 3 | 3 |
| TCGA-V1-A9OH | Methylation_H | 1 | 1 | 1 |
| TCGA-KK-A8IB | Methylation_L | 3 | 3 | 3 |
| TCGA-HC-A9TE | Methylation_L | 2 | 2 | 2 |
| TCGA-XJ-A9DI | Methylation_L | 3 | 3 | 3 |
| TCGA-J4-AATZ | Methylation_L | 3 | 3 | 4 |
| TCGA-EJ-A7NK | Methylation_L | 3 | 3 | 3 |
| TCGA-EJ-5531 | Methylation_L | 3 | 3 | 3 |
| TCGA-KK-A8IC | Methylation_L | 3 | 3 | 3 |
| TCGA-CH-5789 | Methylation_L | 3 | 3 | 3 |
| TCGA-H9-A6BX | Methylation_L | 3 | 3 | 3 |
| TCGA-EJ-A7NJ | Methylation_H | 1 | 3 | 3 |
| TCGA-HC-7817 | Methylation_L | 2 | 2 | 2 |
| TCGA-EJ-A7NG | Methylation_L | 3 | 3 | 3 |
| TCGA-EJ-5521 | Methylation_H | 1 | 1 | 1 |
| TCGA-V1-A9ZI | Methylation_L | 2 | 2 | 2 |
| TCGA-KC-A4BL | Methylation_L | 3 | 3 | 3 |
| TCGA-EJ-A65J | Methylation_L | 3 | 3 | 3 |
| TCGA-HC-A9TH | Methylation_L | 2 | 2 | 2 |
| TCGA-KK-A8I6 | Methylation_L | 3 | 3 | 3 |
| TCGA-KC-A7FA | Methylation_L | 2 | 2 | 2 |
| TCGA-EJ-5503 | Methylation_L | 3 | 3 | 3 |
| TCGA-XK-AAK1 | Methylation_H | 1 | 1 | 1 |
| TCGA-J4-A83J | Methylation_L | 3 | 3 | 3 |
| TCGA-EJ-5506 | Methylation_L | 3 | 3 | 3 |
| TCGA-EJ-A65B | Methylation_H | 1 | 1 | 1 |
| TCGA-HC-7744 | Methylation_L | 2 | 2 | 2 |
| TCGA-EJ-A8FU | Methylation_L | 3 | 3 | 3 |
| TCGA-J9-A8CM | Methylation_L | 2 | 2 | 2 |
| TCGA-CH-5754 | Methylation_L | 2 | 2 | 2 |
| TCGA-EJ-A8FN | Methylation_H | 1 | 1 | 1 |
| TCGA-XJ-A9DQ | Methylation_L | 3 | 3 | 3 |
| TCGA-VN-A88R | Methylation_H | 1 | 1 | 1 |
| TCGA-X4-A8KS | Methylation_H | 1 | 3 | 3 |
| TCGA-HC-A6HX | Methylation_L | 3 | 3 | 3 |
| TCGA-XK-AAJ3 | Methylation_H | 1 | 1 | 1 |
| TCGA-EJ-5515 | Methylation_L | 3 | 3 | 3 |
| TCGA-ZG-A9LU | Methylation_H | 1 | 3 | 3 |
| TCGA-2A-AAYU | Methylation_L | 3 | 3 | 3 |
| TCGA-EJ-7782 | Methylation_L | 3 | 3 | 3 |
| TCGA-V1-A8MU | Methylation_H | 1 | 1 | 1 |
| TCGA-4L-AA1F | Methylation_H | 1 | 1 | 1 |
| TCGA-J4-AATV | Methylation_L | 3 | 3 | 3 |
| TCGA-EJ-5514 | Methylation_H | 1 | 1 | 1 |
| TCGA-ZG-A9LY | Methylation_L | 3 | 3 | 3 |
| TCGA-V1-A9OX | Methylation_H | 1 | 1 | 1 |
| TCGA-XK-AAJR | Methylation_H | 1 | 3 | 3 |
| TCGA-J9-A8CP | Methylation_L | 3 | 3 | 3 |
| TCGA-HC-A8D0 | Methylation_L | 3 | 3 | 3 |
| TCGA-XJ-A83G | Methylation_L | 3 | 3 | 3 |
| TCGA-XK-AAJA | Methylation_H | 1 | 1 | 1 |
| TCGA-EJ-7218 | Methylation_H | 1 | 3 | 3 |
| TCGA-EJ-A7NN | Methylation_L | 2 | 3 | 3 |
| TCGA-YL-A8HK | Methylation_L | 3 | 3 | 3 |
| TCGA-G9-A9S7 | Methylation_H | 1 | 1 | 1 |
| TCGA-V1-A9O7 | Methylation_H | 1 | 1 | 1 |
| TCGA-CH-5788 | Methylation_H | 1 | 1 | 1 |
| TCGA-KC-A7F5 | Methylation_L | 3 | 3 | 3 |
| TCGA-V1-A9ZK | Methylation_L | 3 | 3 | 3 |
| TCGA-EJ-A7NH | Methylation_L | 3 | 3 | 3 |
| TCGA-HC-7738 | Methylation_H | 1 | 1 | 1 |
| TCGA-KK-A6E8 | Methylation_H | 1 | 1 | 1 |
| TCGA-M7-A71Y | Methylation_H | 1 | 3 | 3 |
| TCGA-ZG-A9L1 | Methylation_L | 2 | 2 | 2 |
| TCGA-KK-A8IG | Methylation_L | 3 | 3 | 3 |
| TCGA-XK-AAIV | Methylation_H | 1 | 3 | 3 |
| TCGA-EJ-A46E | Methylation_L | 3 | 3 | 3 |
| TCGA-CH-5743 | Methylation_L | 3 | 3 | 3 |
| TCGA-CH-5766 | Methylation_L | 3 | 3 | 3 |
| TCGA-V1-A9Z9 | Methylation_H | 1 | 1 | 1 |
| TCGA-HC-7213 | Methylation_L | 2 | 2 | 2 |
| TCGA-YL-A8HL | Methylation_H | 1 | 1 | 1 |
| TCGA-YL-A9WX | Methylation_H | 1 | 3 | 3 |
| TCGA-ZG-A9MC | Methylation_H | 1 | 1 | 1 |
| TCGA-FC-A4JI | Methylation_H | 1 | 1 | 1 |
| TCGA-ZG-A9LS | Methylation_H | 1 | 1 | 1 |
| TCGA-VP-AA1N | Methylation_H | 1 | 1 | 1 |
| TCGA-V1-A9OL | Methylation_L | 3 | 3 | 3 |
| TCGA-FC-A66V | Methylation_L | 3 | 3 | 3 |
| TCGA-XJ-A9DK | Methylation_L | 3 | 3 | 3 |
| TCGA-KK-A8IK | Methylation_H | 1 | 1 | 1 |
| TCGA-G9-7521 | Methylation_L | 3 | 3 | 4 |
| TCGA-EJ-A46H | Methylation_L | 3 | 3 | 3 |
| TCGA-HC-8265 | Methylation_L | 3 | 3 | 3 |
| TCGA-KK-A6E1 | Methylation_L | 2 | 2 | 2 |
| TCGA-KC-A7F3 | Methylation_L | 3 | 3 | 3 |
| TCGA-V1-A8WV | Methylation_H | 1 | 1 | 1 |
| TCGA-YL-A9WY | Methylation_H | 1 | 3 | 3 |
| TCGA-Y6-A9XI | Methylation_L | 2 | 2 | 2 |
| TCGA-ZG-A9M4 | Methylation_L | 2 | 2 | 2 |
| TCGA-XK-AAJU | Methylation_L | 3 | 3 | 3 |
| TCGA-ZG-A9N3 | Methylation_L | 2 | 2 | 2 |
